# Agile evaluation including two pragmatic trials on the uptake of a digital screening service

**DOI:** 10.1038/s41746-025-01672-5

**Published:** 2025-05-26

**Authors:** Taavi Tillmann, Andrew Copas, Paul Stokes, Nick Udell, Jo Stead, Jin Lim, Gene Libow

**Affiliations:** 1 Institute of Family Medicine and Public Health, University of Tartu, Tartu, Estonia; 2 Institute for Global Health, University College London, London, UK; 3 Department of Public Health, London Borough of Southwark Council, London, UK; 4 Freelance Software Developer, Dorset, UK; 5 Freelance Service Designer, London, UK

**Keywords:** Disease prevention, Health services, Public health

## Abstract

Digital screening may divert lower-risk persons to lower-cost online screening, or offer higher-risk non-responders a more acceptable alternative. Population-based uptake estimates are lacking. We conducted four studies within four weeks by inviting 1700 Londoners (40–74 years, without cardiovascular disease) to a digital Health Check. A six-arm pragmatic unregistered randomised controlled trial (RCT) tested different Short Message Service (SMS) invitations. Uptake varied from 12% (standard SMS) to 20% (shortest SMS, *P* = 0.009). We tested three sequential reminders (an SMS, a second pragmatic trial [SMS vs postal reminder], and a final SMS). The first SMS reminder increased uptake by +3%. The postal reminder (+7%) was twice as effective as the SMS reminder (+3%, *P* < 0.0001). The “final reminder” SMS added +7%. Altogether, shorter invites, multi-modal reminders, and a “final reminder” all increased uptake. Adding digital care to in person care may raise uptake from 50 to 60%. Agile evaluations can rapidly improve invitation systems.

## Introduction

There are many cost-effective face to face screening services, but they typically achieve only 30–70% uptake^1^. Some nonresponders may prefer other methods, where some data collection, risk stratification or advice is accessed with automated online self-service tools. If these are accessible and acceptable, a mixed-modality programme combining digital and in-person services may increase overall uptake. This has not been previously tested.

New services may be developed linearly, by rigidly following an original plan or specification. Alternatively in *agile development and evaluation*, the plan is repeatedly changed, based on short cycles of iterative feedback. Agile approaches were initially proposed 20–30 years ago for manufacturing^2^, and can be generalised for any team activity^3^. As enshrined in the 2001 Agile Manifesto (https://agilemanifesto.org/), these approaches are particularly applicable for software development, which allows very short lags between deploying service variations and measuring effects (i.e., minutes). Digital service development outside of healthcare (e.g., by Amazon, Netflix or social media companies) typically achieves rapid innovation by running multiple rapid randomised controlled trials (also known as “A/B tests”) sequentially, to iteratively inform rapid development changes^4^. Some have suggested that software with a health or medical application (i.e., e-health, mHealth or digital health) should be developed and evaluated using similar principles and methods^5,6^. However, there are only a handful of examples in the peer reviewed literature that demonstrate how and why to embed the wider agile evaluation environment, and more specifically sequential A/B tests, into digital health service development^7–9^. Methodologically, we explored the feasibility and utility of bringing agile software development and evaluation processes, particularly sequential A/B testing, into public health settings.

Our use case was the English National Health Service (NHS)’s Health Check (NHSHC). This aims to reduce the risk of developing cardiometabolic diseases (stroke, kidney disease, heart disease, type 2 diabetes and dementia) among adults aged 40–74. Since 2009, all eligible residents are invited once per five years for a 20 min in-person check with a primary care nurse or assistant. Much of this time is spent on collecting data on health behaviours, blood pressure, cholesterol and body mass index (BMI), and a little on communicating risk and ways to reduce it. Service delivery is delegated to England’s 150 local authorities (a.k.a. districts or municipalities) who have considerable autonomy in its format^10^.

Southwark Council was the first local authority to design and develop a Digital NHS Health Check Service in 2017. In 2018 Public Health England funded a discovery project to understand limitations of the existing NHS Health Check programme by focusing on digital opportunities^11^; In 2019 Greater Manchester Health and Social Care funded an alpha project to develop and test prototypes service. In 2021/22, Southwark Council funded a beta to build, test, and iteratively improve an end-to-end digital service.

In January 2022, Southwark Council decided to pilot their digital service in the real world. During the evaluation, the digital service itself was frozen to new developments. Conventionally, the invitation process would not have involved any variations, and restricted invites to “early adopter” primary care centres whose staff and patients are more motivated. Instead, we tested multiple variations to the invitation and reminder system (a.k.a. Call/recall system)^12^, on a broader and more representative sample. Specifically, we wanted to see whether our agile process can improve uptake rates, and tested four prespecified hypotheses:

H1 = informing participants about the average time it takes to complete the service (i.e., “20 minutes”) gives greater uptake than the standard invitation.

H2 = the shortest invitation gives greater uptake than the standard invitation. (As secondary analysis to further interrogate the robustness of this, we also tested intermediate-length invitations, which necessitated a multi-arm trial.)

H3 = a printed letter reminder gives greater response than an SMS reminder.

H4 = using the words “final reminder” (as used in financial warning letters) gives a greater response, when compared to the response seen in earlier reminders without this wording.

## Results

Table 1 shows how study participants were similar to those who would otherwise be invited for an in-person NHSHC, except that they were slightly older. This may have arisen as the sample had more “higher risk typical nonresponders” and less “lower risk people” than is found in the general population (Details in “Methods – Two subgroups”). Subsequent randomisation in study 1 achieved approximately balanced allocation of demographic traits across the six trial arms. For wider background, the average 10-year risk of cardiovascular disease was 6.2% among participants on average (4.4% among the lower risk subgroup and 8.7% among the higher risk nonresponder subgroup). Comparable data was unavailable for the wider Southwark population, as many people have never had a cholesterol check.

**Table 1:** Baseline characteristics of the sampling frame, study participants, and six trial arms

	Total	Men	Aged 60–74	Two most deprived area quintiles (as per postcode IMD)	Smokers	Messages not delivered
Southwark population, eligible for a health check	83 670 (100%)	43 631 (52%)	9 309 (11%)	31 153 (37%)	13 387 (16%)	Not Applicable
Participants	1700 (100%)	930 (53%)	318 (19%)	638 (38%)	305 (18%)	308 (18%)
Participants, lower risk subgroup	1019 (60%)	472 (46%)	107 (10%)	413 (41%)	152 (15%)	unknown
Participants, higher risk nonresponder subgroup	681 (40%)	458 (67%)	211 (31%)	225 (33%)	153 (22%)	unknown
Study 1, Randomised to arm-1	283 (100%)	162 (57%)	50 (18%)	102 (36%)	50 (18%)	57 (20%)
Study 1, Randomised to arm-2	283 (100%)	161 (57%)	40 (14%)	110 (39%)	54 (19%)	42 (15%)
Study 1, Randomised to arm-3	283 (100%)	145 (51%)	51 (18%)	99 (35%)	44 (16%)	49 (17%)
Study 1, Randomised to arm-4	283 (100%)	167 (59%)	54 (19%)	111 (39%)	48 (17%)	56 (20%)
Study 1, Randomised to arm-5	284 (100%)	148 (52%)	60 (21%)	110 (39%)	52 (18%)	55 (19%)
Study 1, Randomised to arm-6	284 (100%)	139 (49%)	45 (16%)	113 (40%)	56 (20%)	49 (17%)
Study 2, Sent reminder-1-SMS	1129 (100%)	590 (52%)	206 (18%)	449 (40%)	202 (18%)	7 (0.6%)
Study 3, Randomised to reminder-2-SMS	538 (100%)	274 (51%)	64 (12%)	238 (44%)	92 (17%)	37 (6.8%)
Study 3, Randomised to reminder-2-letter	538 (100%)	312 (58%)	114 (21%)	215 (40%)	93 (17%)	unknown
Study 4, Sent “final reminder” SMS	983 (100%)	521 (53%)	172 (18%)	397 (40%)	172 (18%)	106 (11%)

*IMD* Index of Multiple Deprivation

### Study 1: Which invitation SMS has better uptake?

**Fig. 1:**
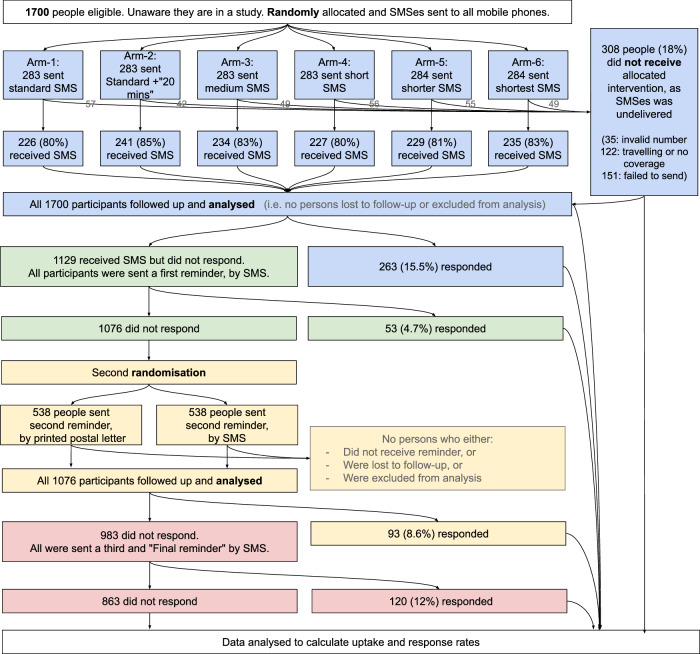
Participant flow throughout the agile evaluation. The four sub-studies are colour coded in sequence as blue, green, yellow, red. “Responders” are defined as people opening a personal hyperlink, entering risk factor data and viewing their results and advice. SMS Short Message Service.

After sending SMS invitations to 1700 participants, the SMS operator found that 308 SMSes were undelivered (Fig. 1). SMSes were not delivered to 15 to 20% participants across six trial arms. Table 2 shows that the standard invitation-1 had the lowest uptake (12%). Uptake was slightly greater with invitation-2 that told participants how the check only takes 20 minutes (14%), but this may have arisen by chance (*P* = 0.38). The shortest invitation had the greatest uptake (20%) and chance alone is unlikely to explain this finding (unadjusted *P* = 0.009; significant at the 5% level after Bonferroni adjustment from 1% for 5 multiple tests). A sensitivity analysis using a per-protocol approach (where participants who did not receive the SMS are excluded from the denominator) found similar results when comparing data between arm-1 vs arm-6, albeit of slightly attenuated effect (OR = 1.43, unadjusted *P* = 0.05).

**Table 2:** Uptake rates following an SMS invitation, before further reminders

Invitation arm	Uptake rate	Odds ratio of responding (95% Confidence Interval)	Unadjusted chi-squared *P*-value, comparing to Standard group
1) Standard	33/283 = 12%	Reference (1.00)	Reference
2) Standard +“takes 20 min”	41/283 = 14%	1.28 (0.76–2.17)	0.38
3) Medium	47/283 = 17%	1.50 (0.91–2.52)	0.12
4) Short	36/283 = 13%	1.10 (0.65–1.89)	0.80
5) Shorter	49/284 = 17%	1.58 (0.96–2.63)	0.08
6) Shortest	57/284 = 20%	1.90 (1.17–3.13)	0.009
TOTAL	263/1700 = 15.5%		

The six trial arms were sent slightly different invitation texts.

### Study 2: Baseline effect of a single SMS reminder

Based on the results of study 1, we then rapidly designed the content of the first reminder by blending content from the best performing invitation (Arm 6: *“You are due an NHS Health Check. Save time and complete it online”*) along with the orthogonal clue that adding *“20* *minutes”* (Arm 2) may potentially increase uptake further. After sending all 1129 non-responding participants a single reminder, 53 additional participants responded. This suggests that a single SMS can generate a “reminder response” of 4.7% among the subset being reminded. From the perspective of the wider cohort of all 1700 people who were initially invited, this reminder increased uptake from a baseline of 15.5% (263/1700) to 18.6% (316/1700) denoting a + 3.1% increase in uptake.

### Study 3: Which reminder modality works best?

Following the first reminder, 1076 participants had still not responded and were randomised. The core emerging themes that arose from the results of studies 1 and 2 (i.e., that short SMSes work best) were then used to rapidly redesign the wording of the second SMS reminder, which was three times shorter than the earlier SMS reminder. Table 3 shows that of those who were reminded the second time by SMS, 4.5% responded. This is very similar to the “reminder response” (4.7%) seen to the first and much longer SMS reminder sent out one week earlier under study 2. This suggests that either a) participants had not yet been saturated with reminders and that subsequent SMS reminders remained similarly effective, or b) saturation was happening, but this was offset by greater effectiveness, as the latest reminder was shorter and more effective than the earlier reminder. The results of a pragmatic trial are shown in Table 2. The odds of responding to the printed letter arm (13%) were 3.10 times greater than seen in the SMS arm, and this increase was unlikely to have arisen by chance (chi-squared *P* < 0.0001).

**Table 3:** Reminder response to the second reminder

Reminder arm	Response rate	Odds ratio of responding (95% Confidence Interval)	Chi-squared *P*-value, unadjusted
A) SMS arm	24/538 = 4.5%	Reference (1.00)	n/a
B) Printed letter arm	68/538 = 13%	3.10 (1.88–5.25)	< 0.0001

In one trial arm (A) this was sent by SMS. In the other trial arm (B) this was sent by a printed letter.

### Study 4: What is the effect of a “final reminder” SMS?

Following the second invitation, 983 participants had still not responded. We then used the results from studies 1–3 (i.e., short SMSes work best) to design a similarly short final reminder. After sending a “final reminder” SMS message to all nonresponders, 120 participants responded. This “reminder response” (12%) is 2.6 times greater than the reminder response seen to the first SMS reminder (4.7%; informal chi-squared *P* < 0.0001) and 2.7 times higher than the reminder response seen to the second SMS reminder (4.5%; informal chi-squared *P* < 0.0001). This increase was unlikely to be driven by message length, but instead by our use of the somewhat threatening wording “final reminder”.

After completing the four studies we were asked by Southwark Council to synthesise a short summary of what the cumulative uptake rates might be (across our two subgroups combined), as participants progress through this newly designed invitation and reminder process (Table 4). We provide these for two theoretical populations. First, cumulative uptake rates on average, across the various service variations that we trialled. Second, cumulative uptake rates that we might expect for a similar population, if the responses we observed in best-performing service variations would generalise and replicate in future samples. To do this, we took the reminder response rates observed in our four studies, and weighted their effect on cumulative uptake according to the sizes of the respective subgroups who received them. For the 18% of people who were not reachable by the initial SMS invitation, we assumed that they were also unreachable by subsequent SMS reminders, and kept them in the denominators to maintain an “intention to treat” approach in the primary analyses. Table 4 left hand columns show how, on average across the invitation variations, 31% of those eligible eventually made it to the results page. If excluding 18% of people whose SMS invitation was not received from the denominator, this would give a per-protocol adjusted uptake rate of 37%. The right hand columns show that the best performing system gave a total uptake rate of 36%. If excluding those who are unreachable, this would give a per-protocol adjusted uptake rate 44% in the right column.

**Table 4:** Anticipated uptakes for synthetic cohorts of 100 people, modelled using outcome data from this evaluation

	Averaging optimal and suboptimal invitation methods		Using optimal invitation methods only	
	Nr of people	Uptake rate	Nr of people	Uptake rate
Eligible	100	ref.	100	ref.
Invited	82	82%	82	82%
Responded to invite	15	15%	20	20%
Responded to reminder-1	+3.1	18%	+2.9	23%
Responded to reminder-2	+5.5	24%	+7.5	30%
Responded to reminder-3	+7.3	31%	+6.3	36%
**Total** **uptake rate**	**31**	**31%**	**36**	**36%**

The left sided columns designate a hypothetical invitation system that averages the effect of short and long invitations, and SMS/printed reminders, in the same proportions as we did in this study. The right sided columns designate a hypothetical invitation system that uses the shortest invitation SMS and a postal reminder-2. We assume response rates to each reminder are as seen in our study.

Uptake varied between the two subgroups enrolled into the study. Among higher risk people who had not previously taken up the in-person offer, uptake was markedly greater than zero, with 18% (103/570) now responding across all service variations. If the best performing service were applied here, we would expect 21% of historic nonresponders to now respond. Among lower risk people, uptake was twice as high (426/1130 = 38%) across all service variations.

In addition to the primary outcome of uptake, the digital health check subsequently offers behaviour change support. After viewing their results, participants can select priorities (e.g., quit smoking), identify their barriers to change, and select support from a list of automatically personalised options (e.g., local smoking cessation clinics). Of those who reached the results page, 57% (*N* = 302/529) selected priorities and viewed personalised advice. Secondly, of those who made it to the results page, 46% (*N* = 243/529) received a non-urgent referral to their GP, with details to follow-up a potentially actionable risk factor (e.g. smoking, obesity, systolic blood pressure >140 mmHg, potentially eligible for statins or diabetes prevention programme).

## Discussion

We exploited a natural opportunity when piloting a novel digital service, to vary multiple dimensions of the invitation system in an agile manner within just three weeks. Four approaches appeared to increase uptake. First, the shortest invitation SMS (which gave participants only one option) doubled the uptake rate; second that three subsequent reminders approximately doubled uptake further, with no evidence of saturation; third that using a mixture of SMS and printed modalities gave higher uptake than SMS alone; and fourth that using the word “final reminder” gave an unexpectedly large increase in uptake. Half of those who made it to the results page had an actionable risk factor, warranting a referral to primary care. Altogether, the digital offer may be an acceptable alternative for around one-fifth of those who historically disengage from in-person care.

More broadly, this set of findings exemplifies how an “agile development and evaluation” approach may be a fast and efficient way to create innovation and new knowledge in digital health services. By our definition, outcome data from one study must influence the design of a subsequent study, and both of these have to be conducted on the same population within 12 months of one another. We encourage others to consider similar approach, particularly when there is a short delay (i.e. hours or days) between deploying a service variation and accessing data on outcomes.

Consistent with our findings, previous studies have also found SMS reminders to increase cumulative uptake. This magnitude has typically ranged from +3% for one SMS, to +9% of the total eligible cohort when using as many as 15 SMS reminders per patient^13–17^. Our cumulative effect of +14% is higher than previously reported. This may be partly explained by our use of the words “final reminder”, which has not been tested before. Secondly, after varying the reminder modality to a trio package (SMS-postal-SMS), the cumulative effect of this package of reminders on uptake rose to +17%. To the best of our knowledge, this is the first randomised analysis that demonstrates how mixed-modality reminders are superior to single-modality reminders. Finally, it is possible that when the service being taken up is exclusively digital, then baseline uptake rates may be lower, making it easier for reminders to generate higher uptake. To the best of our knowledge, previous studies have not described uptake rates for exclusively digital screening services.

Our work has several limitations. First, we did not publish our prespecified protocol for studies 1–2 (and we did not formulate protocols for studies 3–4 in advance, due to the explicitly agile process of formulating these based initial results). This increases the risk of bias from selective reporting. The risk is partly offset by the type of primary outcome (uptake), which is difficult to measure or analyse in multiple ways. Nonetheless, this limitation alone might downgrade the quality of our two trials into “intermediate risk of bias” as per the Cochraine Risk of Bias II tool^18^. Second, small sample sizes left some trial arms partly imbalanced. For example, the proportion of undelivered SMSes varied from 15 to 20% across the six arms, making it possible for such variation to cause differences in uptake. However, our per-protocol analysis gave similar results, making it unlikely for such phenomena to drive the large differences in uptake we observed. Third, our statistical approach did not fit formal dose-response curves that may more efficiently utilise outcome data from the middle of the dose curve. Fourth, after having gathered all the data, it took us 2 years to submit these results for peer review. This was partly because once the real-world service improvement project had finished, we were unable to identify discrete funding to support the subsequent academic publication of its findings.

Strengths of this study include its real-life, routine and pragmatic setting, that participants were blinded and unaware of the study, and how the outcome data had no missingness and is difficult to manipulate. The real-life and representative setting is particularly uncommon in trials of digital health interventions. However, our participants were slightly older. This reflects how we over-enriched the sample with persons of elevated cardiovascular risk. Second, we excluded approximately 2% people who did not have a mobile phone number on their GP record, so the likely true population-based uptakes are marginally lower than what we report. Overall, we believe that these results should replicate in most high-income settings where reliable phone numbers are available for most of the population, these have been used by legitimate healthcare providers to interact with patients, and where cardiovascular diseases are perceived as being important among the general public.

Service developers could potentially develop the NHS digital health check in two directions: first, to shift lower risk people away from in-person care towards digital care that has considerably lower costs. Second, digital care could expand the reach of the programme beyond the current ceiling of 50% achieved by in-person care^3^. We saw meaningful uptake (18%) among the subgroup of higher risk nonresponders. As they had previously declined in person care, even low levels of uptake are both difficult to achieve and potentially valuable. By extrapolation, population-based uptake may rise from 50% to at least 59% (if not to 60.5% if using the best performing reminder system), if the digital offer were added as a second line measure for those who decline in person care.

Future research could consider agile evaluations of the downstream process of behaviour change and medication usage, once such needs are identified. Subsequently, definitive trials could establish whether cardiovascular screening is effective^19^, and/or whether digitally-blended services are more cost-effective than in-person services. Finally, cancer screening programmes may also trial sending a higher volume of shorter reminders across a mixed range of modalities, and use terms like “final reminder” once interval times have become dangerously long.

To conclude, conventional face to face health checks typically achieve 50% uptake. If nonresponses are offered additional digital care, a further +10% would uptake the digital service based on the best reminders that we trialled. This suggests that uptake rates of up to 60% may be possible when combining digital and face to face care. Secondly, if lower risk residents are channel shifted, whereby digital care replaces face to face care, then a slight decline in uptake (38% for online care vs 50% for face to face care) may be worth the cost savings, if few of these newly nonattending low risk users have actionable risk factors, however these trade-offs need further study.

## Methods

### Theory background

Pragmatic trials of healthcare services typically have all the features (including power and sample size) clearly specified before the intervention starts. When designing our wider evaluation, an absence of previous literature prevented us from anticipating what uptake rates we might see. This made it hard to propose reliable arm size(s) and number(s) for each of the studies a priori. Instead, we applied and further developed a more flexible approach that might be called *“agile evaluation”*. Into this we nested pragmatic trials and other evaluation tools. For wider background, this approach builds on three existing theoretical concepts. First, *“megastudies”* test multiple competing arms against one another, however do not modify the protocol or learn over time^20^. Second, *“adaptive trials”* can modify the intervention, as its implementation unfolds. However, their interim findings are not thought of as primary outcomes themselves^21^. Third, a broader process model (partly inspired by an earlier *“IDEAS model”*)^22^, called the *“mHealth Agile Development & Evaluation Lifecycle”* has merged agile software development processes along with the four phases of conventional pharmaceutical evaluation^23^. This is difficult, as software development is less risky and culturally more focused on speed and change, while healthcare is more risk-averse with a cultural focus on safety and slowly following detailed pre-specified plans. Nonetheless, according to this lifecycle model, our evaluation is nested within the “Phase 3: Clinical Trial Evaluation” stage. Here, A/B testing is often encouraged but there is limited guidance as to how to exactly do this. These authors had speculated that within Phase 3, “novel study designs may also be better suited to evaluating mHealth products than standard randomised trials”, which we now further develop and elaborate.

### Theory extension – agile evaluations for digital health care

Our work builds on this relatively short and fragmented theoretical literature, by applying and further developing the *“Agile evaluation”* concept, to make it suitable for digital health research. We propose that this should meet two conditions: first, that at least two A/B studies (which strive for many of the quality standards of randomised pragmatic trials, however recognise that full imitation may not always be feasible on account of the need for speed) are conducted in sequence on the same population within 12 months; and second, that outcome data from the first studies are used to influence the design of the subsequent studies. Future agile evaluations may be particularly suitable for Phase 3 (a.k.a. post-beta) digital health service development and evaluation projects, where A/B testing is commonly indicated. To the best of our knowledge, very few “*agile evaluations”* (or *“agile sequence of trials”*) have previously been published in digital health settings^7–9^.

Conventional evaluations of health services typically report outcomes on, and seek to optimise, a single process. However, when patients enter a clinical guideline or care pathway experience, they usually undergo dozens (if not hundreds) of plausibly optimizable process steps. We hypothesised that uptake may change after 1) informing participants that it takes 20 min to complete the check; 2) shortening the length of the SMS invite; 3) modulating the delivery of the reminder across SMS and printed letter format; and 4) using the words “final reminder”. We speculate that this agile evaluation process we propose would be particularly suited to other clinical settings where patients undergo a handful of sequential steps, and where a small number of people or organisations have considerable power in varying their nature. For example, it may be particularly applicable when considering, varying and studying how and when to communicate with patients (by varying the quantity; modality; length, timing and emotional valence of such communications). As such, when the object of study is the *wider communication package*, it may be more efficient and informative to apply agile evaluation in studying a wider set of linked steps, as opposed to the slower approach of isolating each step for a standalone study.

### Design

The study aims were prespecified with the funder in a contract signed in January 2022. We had 3 months to design, deliver and evaluate variations of the invitation system. Such limited time is suboptimal for conventional research, however is common in public health practice. We hope that this project could inspire others tasked with real-world implementations, to consider augmenting their delivery plans so that these might create additional value also to the research literature. We prespecified 4 hypotheses (detailed at the end of the Introduction section) and the detailed design of studies 1–2. Due to time constraints, we did not register these intentions before launch, which is a major limitation. After analysing the outcome data from studies 1–2 (on informing participants that it takes 20 min to complete the check; and on shortening the length of the SMS invite), we had one week to re-read our remaining hypotheses and propose the design and detail of study 3. After analysing data from study 3 (on modulating the delivery of the reminder across SMS and printed letter format), we had one week to re-read our hypotheses and propose the design and detail of study 4 (on using the words “final reminder”). The implementation of the study encountered no deviations from plan that might otherwise arise from unanticipated factors in the trial context, delivery or fidelity.

### Setting

The study was conducted in the inner-London Borough of Southwark, with a population of 317,000. Southwark has 43 primary care practices, of whom Southwark Council selected 9 to participate in the pilot. Thus the pilot was conducted among a wider population of up to 66,000 citizens. Primary care data on all 66,000 citizens was analysed to determine eligibility. As most participants accessed the digital service from their mobile phone (likely while at home or work), we consider the study setting as the pre-primary care level.

### Participants

Eligibility criteria were identical to the routine in-person NHSHC service (i.e., aged 40–74, registered with a Southwark General Practitioner (GP), not had an NHSHC within the last 5 years, no past medical history of stroke, heart disease, heart failure, diabetes, kidney disease, hypertension, atrial fibrillation, or hypercholesterolaemia in the GP record)^10^. We assumed that this data was never missing in the GP record. Participants had to have at least one mobile phone number recorded in their GP record at the time of enrolment, and by applying this criterion, we had to exclude 2.06% of participants. (After enrolment, it transpired that 18% of participants with a recoded phone number were unable to receive or open the Short Message Service (SMS) we sent them, however we still kept these participants in the main analysis.) Altogether, this identified 17,512 eligible participants. Southwark Council decided that this number was too large to invite them all into the pilot. In this evaluation, we only monitored people to see if they engaged with and completed the online tool, and as thus the intervention was explicitly designed to exclude those who were digitally illiterate. However, the invitation letter and landing page offered non-digital service alternatives (“Alternatively, for a face-to-face appointment call X”), the use of which is beyond the scope of this article.

### Two subgroups

Among the eligible participants we did not select a random population-based sample. Instead, we used digital care as an opportunity to nudge the healthcare system towards a more personalised and predictive delivery model, by defining two subgroups where digital services may be most useful. On the one extreme, digital care may *augment* existing face to face care, for a small group of high-value patients who have previously disengaged. This increases costs, and may increase net cost-effectiveness if true need can be accurately predicted and subsequent uptake is high^24^. On the other extreme, digital care may *replace* face to face care, particularly for lower value patients who previously over-engaged. This lowers costs (a major argument for securing political buy in for otherwise complex innovative projects) and may increase cost-effectives if overuse can be accurately predicted^25^. Accordingly, one third of the sample included people whose electronic healthcare record indicated that they may be eligible for subsequent preventative interventions (i.e. predicted 10-year risk of cardiovascular disease based on QRISK3 > 10% and/or diabetes based on QDiabetes > 5.6%) AND who had not taken up an offer of an in-person health check 5–10 years before this study^26,27^. This subgroup (called “higher risk nonresponders”) may benefit most from additional and augmented digital care. Second, we counterbalanced this with two-third of the sample including people whose predicted QRISK3 risk < 10% and whose QDiabetes risk < 5.6% (regardless of historic service attendance). In this subgroup (called “lower risk”), around half would conventionally use in-person health check services, but future funders may prefer to divert some funding towards digital care to lower costs and increase cost-effectiveness. We used participants’ postcode address to generate a proxy of their neighbourhood’s deprivation status. This has partial correlation with cardiovascular and diabetes risk, however neighbourhood deprivation is an independent feature, present in both of the two subsamples described above.

### Delivery

The total sample size (*N* = 1700) was determined by Southwark Council, based on how many residents they were comfortable exposing novel service variations to without incurring excessive reputational risk (incl. fear of overburdening GP clinics with unanticipated demands). Accepting this, we did not conduct an a priori power calculation. Automatic and mandatory enrolment took place within a closed and private database. This agile evaluation was part of a real-world service development project operating within the parameters of national legislation that requires all eligible residents to be invited into a health check. Accordingly, invited participants were unaware of the evaluation of the interventions, were fully blinded, were not asked for their consent, and all were automatically enrolled prior to randomisation. Participants were unaware of their assigned intervention, limiting social desirability bias. Participants provided their own binary outcome data automatically (i.e., completing or not completing the online health check), so outcome measurement was blinded with respect to their assigned intervention. The intervention was delivered by a digital automated software system, not human beings. Southwark Council commissioned an IT partner (QMS Ltd) to build, host and run the digital service. They accessed data from primary care records, identified eligible people and randomly selected individual participants (with a ratio of 1:2 from the aforementioned two subgroups) until *N* = 1700 was reached.

### Ethical considerations

The key service stakeholders (Southwark Council, local primary care practices, and IT providers) created and launched service variations that are all compliant with their legal and clinical governance guidelines. Participants were unaware of their involvement in this study, since we decided to waiver the conventional need to obtain informed consent. This is conventional research practice for studies that measure the uptake of screening services, as other designs undermine the pragmatic nature and generalisability of the findings. Participants were not asked to provide any new data which they would not otherwise provide during their usual care. We analysed pseudonymised data. As per the UK Health Research Authority guidelines (hra-decisiontools.org.uk/ethics/), since we did not study a medicinal product or device, did not involve radiation, embryos, tissues, DNA or transplantation, did not recruit patients via healthcare service providers, did not collect new data for research purposes, and did not target vulnerable patients, then this study was exempt from NHS Research Ethics Review. Otherwise, the study investigators performed this in accordance with the Declaration of Helsinki.

### Interventions

Person-specific encrypted URL links and associated information were sent by a third provider (iPlato Ltd.) who sent out invitations and reminders to participants. The digital service providers harboured an organisational space one level higher up from a primary care practice. All participating GPs signed data sharing agreements and informed their front-line staff about the project in case of enquiries, with none disagreeing. No proactive in-person care was delivered. The questionnaire was entirely digital.

Most participants did not know at least one of their biometric measurements (either blood pressure, cholesterol, BMI and in case of heightened diabetes risk HBA1C). All were offered the chance to collect this through home testing kits or at walk-in facilities in fitness centres and pharmacies, however very few took this up.

Potential clustering by family or GP practice was unlikely, as the study enrolled a small proportion of the target population. No other patient-facing communication or marketing occurred. We did not detect any potential ‘contamination’, such as incorrect SMSes being sent to participants.

### Randomisation and masking

In study 1, computer-generated random numbers were used to allocate 1700 individual participants to six parallel arms with a ratio of 1:1:1:1:1:1 on 29th March 2022. No restriction, blocking or stratification was used in the randomisation process. We did not change or update the pre-planned allocation rules. The allocation sequence was created by two IT companies (North and South Southwark GP federations) who automatically enrolled and assigned all eligible participants. Participants (who also provided their own outcome data) were fully blinded, but those doing the analysis were aware of which arm received which content, to simplify project administration. In arm-1, participants were sent the standard SMS invitation that had been used in an earlier service prototype. In arm-2, participants were additionally told about the duration of the online check to test hypothesis 1. Hypothesis 2 was tested by comparing the shortest invitation (arm-6) against the standard invitation (arm-1). We further tested invitations of intermediate length (arms 3–5) to explore the dose-response curve, however recognised that these were underpowered for definitive testing. All SMSes began with the name of the primary care provider, and “Dear [First Name]”. All SMSes ended with a person-specific unique encrypted URL weblink.

*Arm-1 (Standard): You are due an NHS Health Check to assess your heart disease and diabetes risk. Save time and complete it online, or call for a face-to-face appointment*.

*Arm-2 (Standard +“takes 20* *min”): You are due an NHS Health Check to assess your heart disease and diabetes risk. Save time and complete it online in 20* *min, or call for a face-to-face appointment*.

*Arm-3 (Medium): You are due an NHS Health Check. Save time and complete it online, or call for a face-to-face appointment*.

*Arm-4 (Short): It’s time for your NHS Health Check. Complete it online, or call for a face-to-face appointment*.

*Arm-5 (Shorter): Complete your NHS Health Check online, or call for a face-to-face appointment*.

*Arm-6 (Shortest): You are due an NHS Health Check. Save time and complete it online*.

Other than varying the content of the SMS, participants in all arms had exactly the same experience with their subsequent care. All groups had identical recruitment, allocation, invitation and follow-up dates and hours (to the nearest 5 minutes).

“Responders” were prespecified as people opening a personal hyperlink, entering risk factor data and viewing their results and advice. This best approximates previous metrics of uptake measured in the in-person service. Partial response (e.g., dropping off half-way) was coded as equivalent to “no response”.

In study 2, on 31st March 2022, we sent an SMS reminder to all 1129 participants who had not responded. It read, *“You are due an NHS Health Check. Save time and complete it online in about 20* *min. Already started? Click this link and you will be given the option to skip forward.”* This established a baseline parameter to test hypothesis 4.

**Fig. 2:**
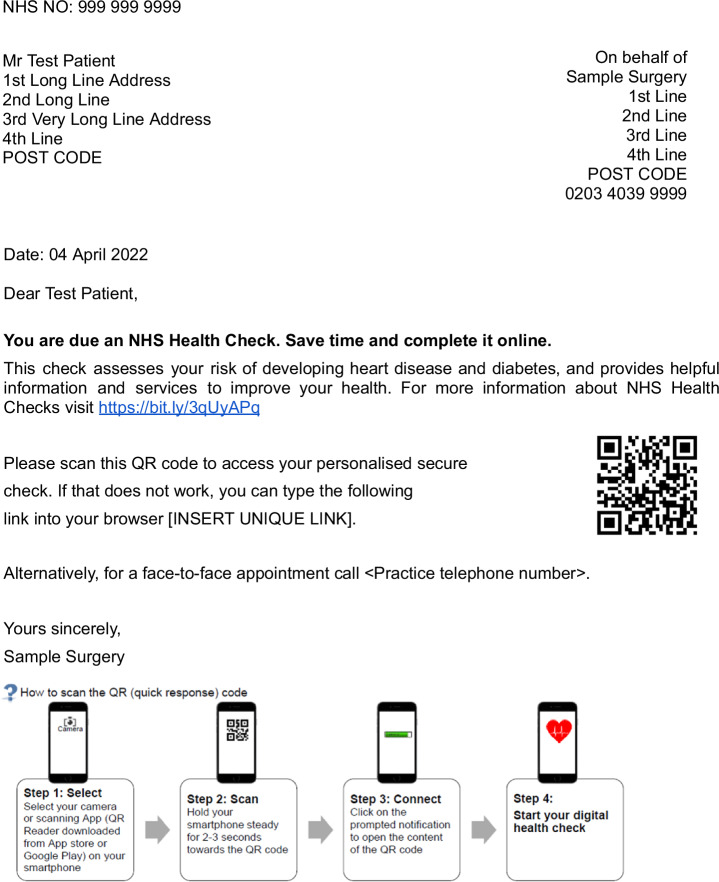
Example of a printed reminder letter. This was sent to a random half of those people who had successfully received an SMS invitation and an SMS reminder, but despite this had not completed the Digital Health Check service.

In study 3, on 7th April 2022 we randomly allocated all 1076 participants, who had not responded, to two parallel arms. This tested hypothesis 3. Computer generated randomisation was again used, the allocation ratio was 1:1 and no restrictions were applied. In one arm, participants were sent an SMS that read, *“This is your reminder to complete your NHS Health Check online”*. In the second arm, participants were sent a printed letter as shown in Fig. 2.

In study 4, on 21st April 2022 we sent a final SMS reminder to all 983 participants who had not responded, to test hypothesis 4. The text read: *“This is your final reminder to complete your NHS Health Check online.”*

Participants who opened the link at any point were taken to a website that was formatted according to the device resolution (commonly a smartphone, occasionally a computer). The landing page was co-branded with “NHS” and “Southwark Council” logos, with no private companies shown. Participants completed a standardised questionnaire about their cardiovascular risk factors. Some participants received further questions (e.g. about binge drinking or hormone replacement therapy), depending on their answers to previous questions. If participants did not know a biometric measurement, an age-sex adjusted national average was temporarily used. Such participants were offered the chance to measure this with either a self-sampling finger prick blood test kit posted to their home, or at a kiosk, leisure centre or pharmacy. The digital service ends with a personalised summary about the users results and recommendations (also sent to their GP clinic). For the purposes of this paper, we define “responders” as participants who successfully made it to this page (regardless of whether they knew their biometric parameters or not).

For wider context, we describe the downstream service thereafter. About 50% of responders are lower risk, and are given reassurance and personalised signposting to local services that can help maintain their health. About 40% of responders are medium risk, and are advised to change a specific health behaviour, and given personalised signposting and self-referral methods to achieve this. About 10% of responders are clinically high risk, and are advised to book a non-urgent appointment with their GP clinic. The supplementary materials provide detailed screenshots of the service.

### Statistical analysis

We define “uptake” as the proportion of people opening a personal hyperlink, entering risk factor data and viewing their results and advice, divided by the total number of people who were initially invited into the digital service (*N* = 1700). In the digital development community, this metric is sometimes called “completion”. Consistent with an intention-to-treat approach, this includes people who were sent, but who did not receive, the invitation. We also report a secondary metric in studies 2, 3 and 4 called “reminder response”. This uses the same numerator, but the denominator includes only participants who were randomly allocated to receive this specific reminder. Outcome data was not missing for any participant and its definition was not changed during the evaluation.

Pearson’s Chi-squared test with Yates’ continuity correction was used to test hypotheses, based on two-sided testing. Odds ratios were calculated with 95% confidence intervals using Fisher’s exact test. The conventional 5% significance level was used in all studies, except for the five pairwise comparisons of each amended reminder to the standard reminder in Study 1. Here a Bonferroni adjustment was applied resulting in a significance level of 5%/5 = 1% (the *P*-values themselves were not adjusted). In Study 4, we compared the reminder response of the final reminder to earlier reminders on a partly overlapping sample separated instead by time. We applied the Chi-squared test for this, which usually assumed independent and nonoverlapping samples. As such, we consider such testing to be informal and indicative only. No data was collected to investigate any potential harms, and we did not notice any unintended effects. Other than breaking the whole evaluation into four studies, there were no further interim analyses. We did not perform subgroup or adjusted analyses. Although qualitative data was used in other phases of this wider project, it was not used to inform this evaluation. The drafting of this article was informed by the CONSORT guidelines and four of its extensions (CONSORT-EHEALTH, multi-arm trials, and adaptive designs)^28–31^, as detailed in the Supplementary information.

## Supplementary information

Screenshots of the service

CONSORT extensions

## Data Availability

We support data sharing in principle. However, given the multiple data controllers and data processors involved, the practicality of sharing the data used in this project is very difficult. Proposals may be submitted and in exceptional circumstances jointly approved by Jin.Lim@southwark.gov.uk and taavi.tillmann@ut.ee, within 5 years following article publication. The code for the digital service's software is open source and is built with the UK Government Design Principles, so that other public health organisations can easily use and build on what was created.
