# Supplementary material for: Agile evaluation including two pragmatic trials on the uptake of a digital screening service
Source: NPJ Digit Med. 2025 May 26;8:310. doi: 10.1038/s41746-025-01672-5 (PMC12106754; doi:10.1038/s41746-025-01672-5)
Supplement: Supplementary file 1 — Screenshots of the service [file 41746_2025_1672_MOESM1_ESM.pdf]

Southwark's Digital NHS Health Check

Screenshots of service

The code for this software is open source, and created according to the UK Government Design Principles

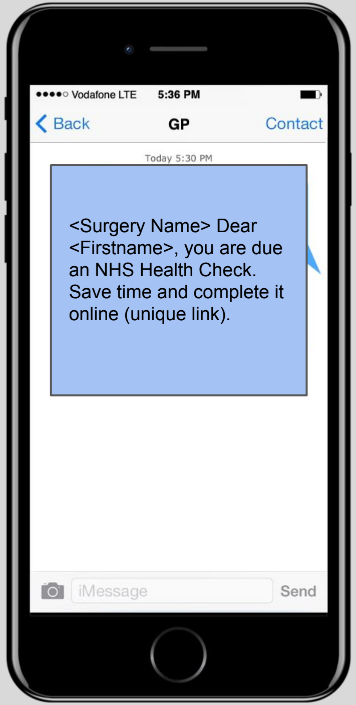

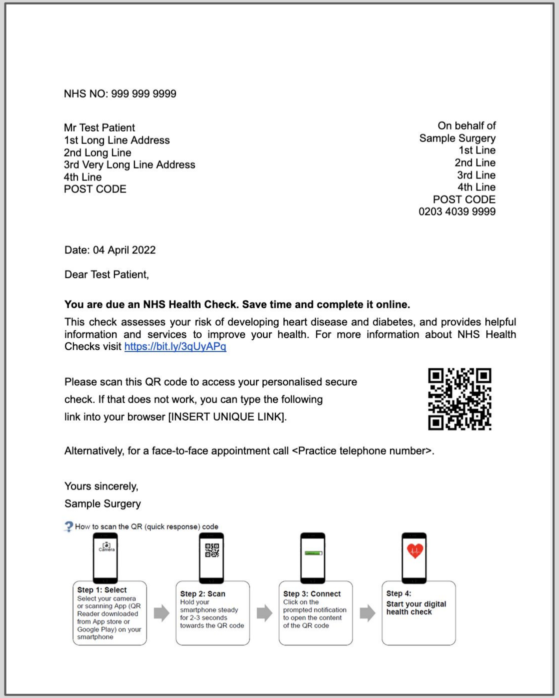

NHS NO: 999 999 9999

Mr Test Patient
1st Long Line Address
2nd Long Line
3rd Very Long Line Address
4th Line
POST CODE

On behalf of
Sample Surgery
1st Line
2nd Line
3rd Line
4th Line
POST CODE
0203 4039 9999

Date: 04 April 2022

Dear Test Patient,

You are due an NHS Health Check. Save time and complete it online.

This check assesses your risk of developing heart disease and diabetes, and provides helpful information and services to improve your health. For more information about NHS Health Checks visit https://bit.ly/3qUyAPq

Please scan this QR code to access your personalised secure check. If that does not work, you can type the following link into your browser [INSERT UNIQUE LINK].

Alternatively, for a face-to-face appointment call <Practice telephone number>.

Yours sincerely,
Sample Surgery

How to scan the QR (quick response) code

Step 1: Select Select your camera or scanning App (QR Reader downloaded from App store or Google Play) on your smartphone

Step 2: Scan Hold your smartphone steady for 2-3 seconds towards the QR code

Step 3: Connect Click on the prompted notification to open the content of the QR code

Step 4: **Start your digital health check**

Invitations: SMS and letters

Each citizen gets an initial SMS invitation, an SMS reminder 1 week later, a letter invitation two weeks after the initial SMS, and a "final" SMS reminder 3 weeks after the initial SMS. Each message contains a unique encrypted link that is specific to the individual.

NHS

NHS Health Check

The NHS Health Check is a free checkup of your heart and circulatory health

You have been invited by your GP clinic to undertake this remote medical assessment. Your results will automatically be shared with your GP clinic.

Use this service to:

- understand your risk of developing heart and circulatory diseases, as well as Type 2 diabetes
- understand your mental wellbeing
- find personalised information and support that can help you lead a longer, healthier life
- access clinical follow-up and support services, if needed
- on average, this service takes 20 minutes to complete

Start Now >

This new remote service is an additional option to the current face-to-face service. You can also access this service via your local GP Clinic (opens in a new tab).

- ▶ How will the NHS Health Check help me?
- ▶ What follow-ups might I need?
- ▶ What support is available to help me?
- ▶ Tell us about your health measurements or get new ones

We will process your data securely and legally; further information is available in the full privacy statement.

- ▶ Who is eligible for the NHS Health Check?

Disclaimer: This service measures your future risk of heart and circulatory diseases and Type 2 diabetes. It does not provide a medical diagnosis of Type 2 diabetes or heart and circulatory diseases. If you feel unwell you should seek clinical support from a healthcare professional by contacting your GP clinic, NHS 111 or, if it is an emergency, 999.

Privacy Cookies Terms and conditions Accessibility Submit feedback

OGL
All content is available under the Open Government Licence v3.0, except where otherwise stated

Southwark Council
southwark.gov.uk
Southwark Council

Start page

Once the service user clicks the link in the SMS or uses the QR code in the letter, they arrive to the start page, which explains what the service is.

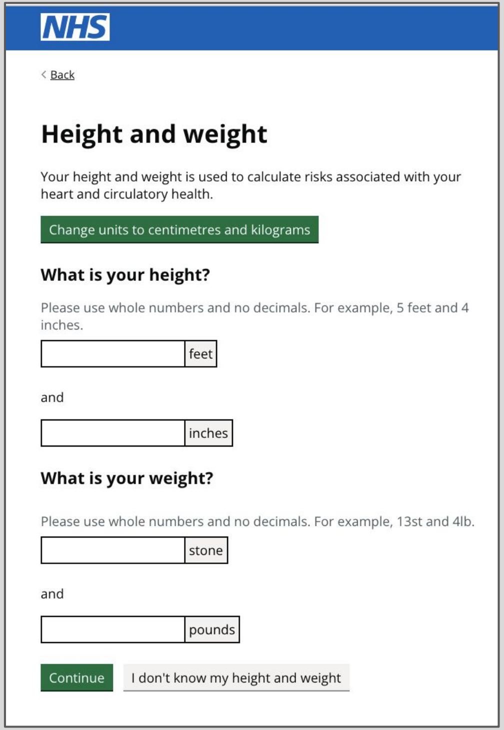

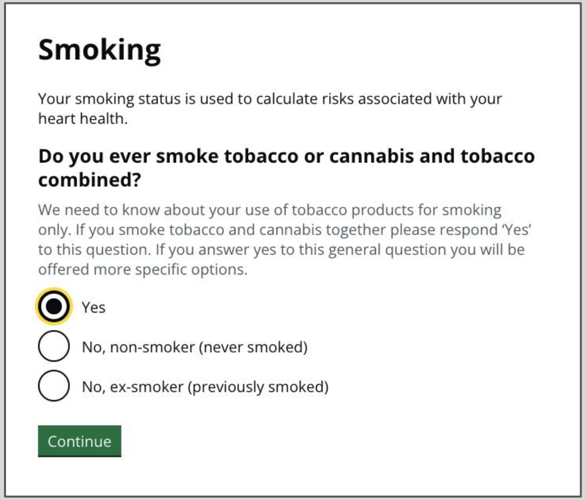

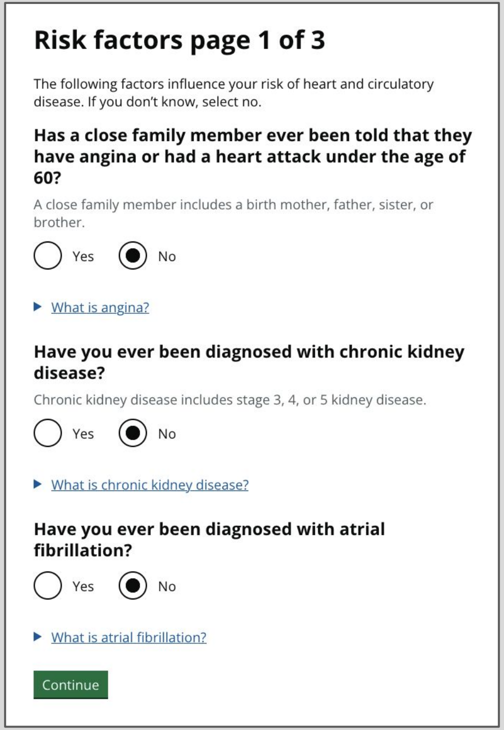

Questions: summary

The experience begins with questions about the service user's biometrics and health beahviours, including: height and weight, gender identity and sex assigned at birth, ethnicity, smoking, alcohol, physical activity, diabetes risk factors, blood sugar, other risk factors, blood pressure, cholesterol.

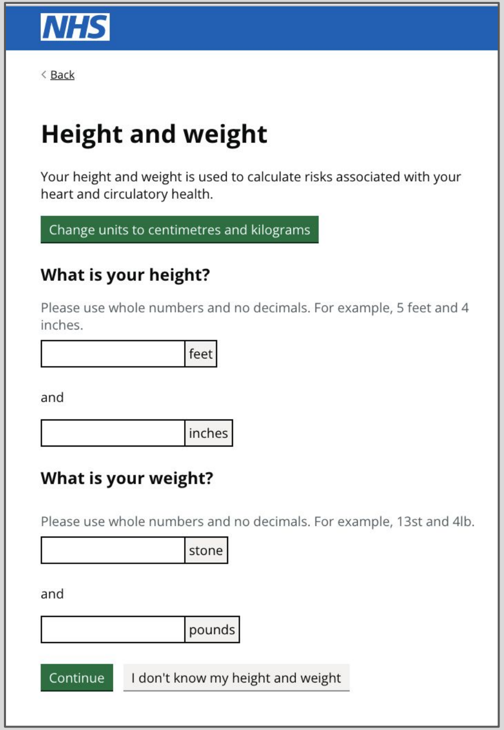

Questions: height and weight

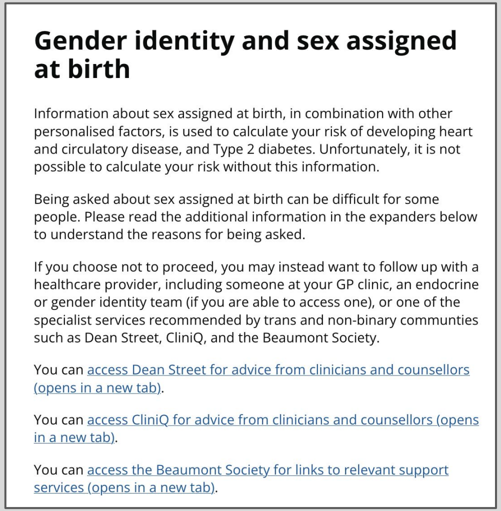

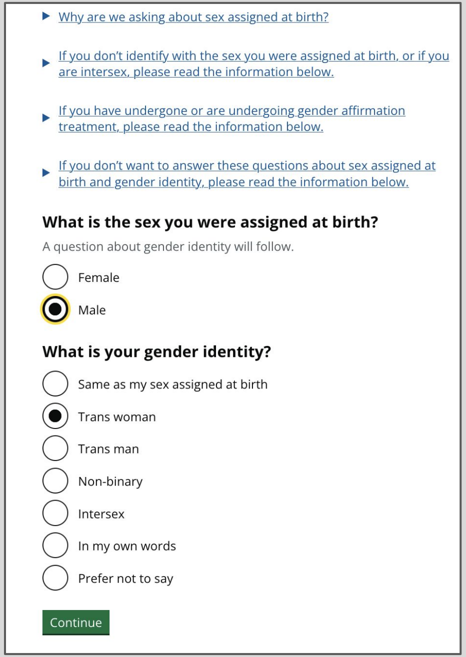

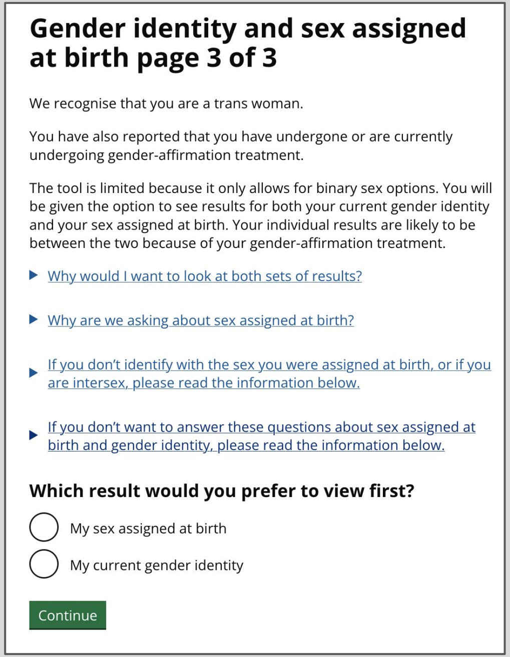

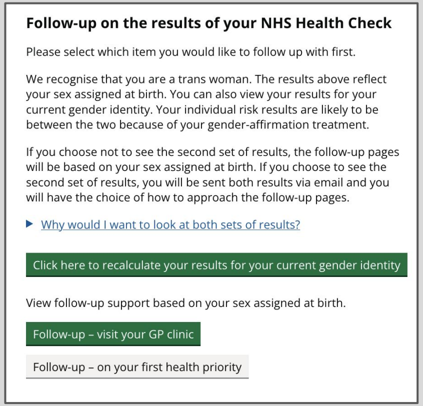

Questions: Gender identity & sex assigned at birth

We built new pathways to make the service more inclusive. We provide more contextual information and create space for service users to choose their own path and be able to explore multiple paths through the service.

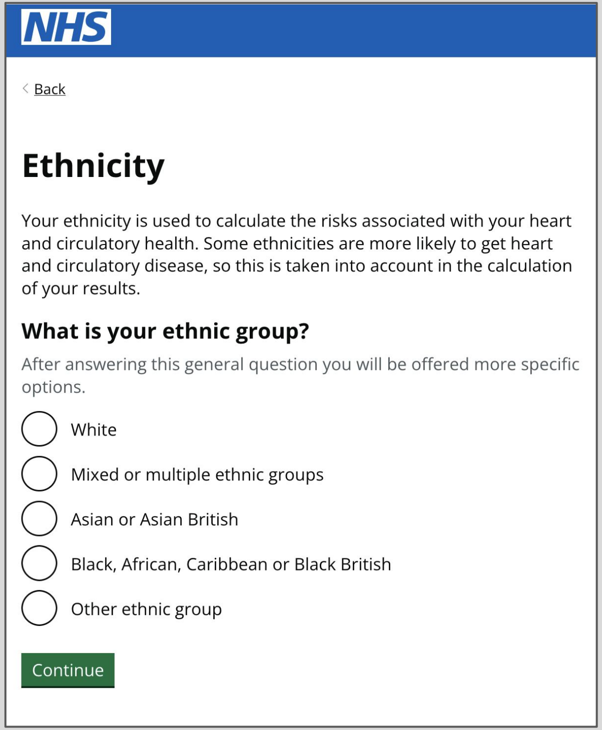

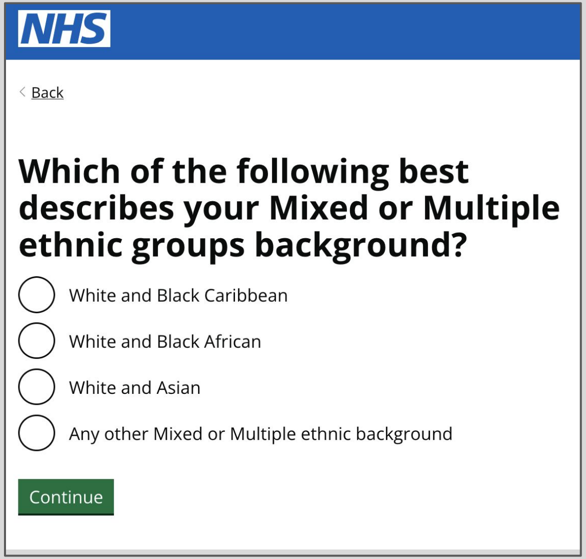

Questions: ethnicity

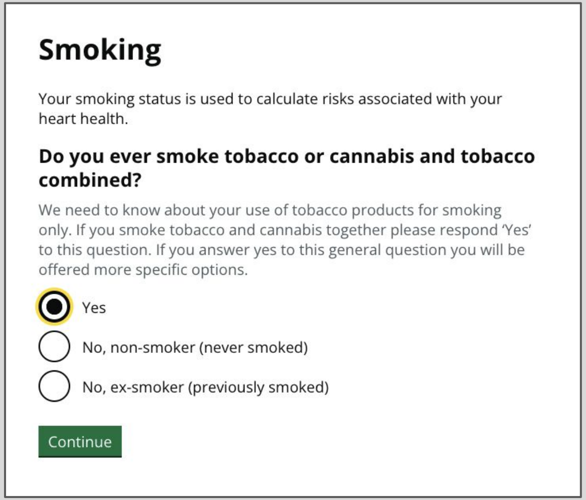

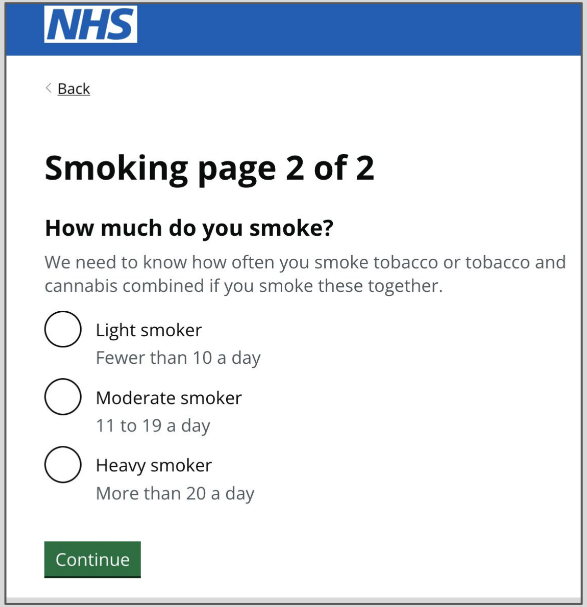

Questions: smoking

NHS

‹ Back

Alcohol

Your drinking status is used to calculate the risks associated with your heart health.

Do you drink any alcohol?

If you answer yes to this general question you will be offered more specific options.

◯ Yes ◯ No

Continue

Alcohol page 2 of 7

How often do you have a drink containing alcohol?

◯ Less than monthly
◯ Monthly
◯ 2 to 4 times per month
◯ 2 to 3 times per week
◯ 4 times or more per week

Continue

Alcohol page 3 of 7

How many units of alcohol do you drink on a typical day when you are drinking?

◯ 1-2
◯ 3-4
◯ 5-6
◯ 7-9
◯ 10+

▶ How many units are in alcoholic drinks?

How often have you had 6 or more units of alcohol on a single occasion in the last year?

◯ Never
◯ Less than monthly
◯ Monthly
◯ Weekly
◯ Daily or almost daily

Questions: alcohol - full AUDIT-C

NHS

< Back

Physical activity page 1 of 4

Your physical activity status is used to calculate risks associated with your heart health.

What type and amount of physical activity is involved in your work?

- ○ Mostly sitting
- ○ Mostly standing or walking
- ○ Definite physical activity
- ○ Vigorous physical activity

or

- ○ I am not in employment

Continue

Physical activity page 2 of 4

During the last week, approximately how many hours did you spend on each of the following activities?

Physical activity

For example, swimming, jogging, aerobics, football, tennis, gym workout. This question does not include walking, gardening or housework. There will be detailed questions about these activities on an upcoming page.

- ○ None
- ○ Some but less than 1 hour
- ○ Between 1 and 3 hours
- ○ 3 hours or more

Cycling

Include cycling to work and for recreation.

- ○ None
- ○ Some but less than 1 hour
- ○ Between 1 and 3 hours
- ○ 3 hours or more

Physical activity page 3 of 4

During the last week, approximately how many hours did you spend on each of the following activities?

Gardening or DIY

- ○ None
- ○ Some but less than 1 hour
- ○ Between 1 and 3 hours
- ○ 3 hours or more

Housework or childcare

- ○ None
- ○ Some but less than 1 hour
- ○ Between 1 and 3 hours
- ○ 3 hours or more

Continue

Questions: physical activity - full GPPAQ

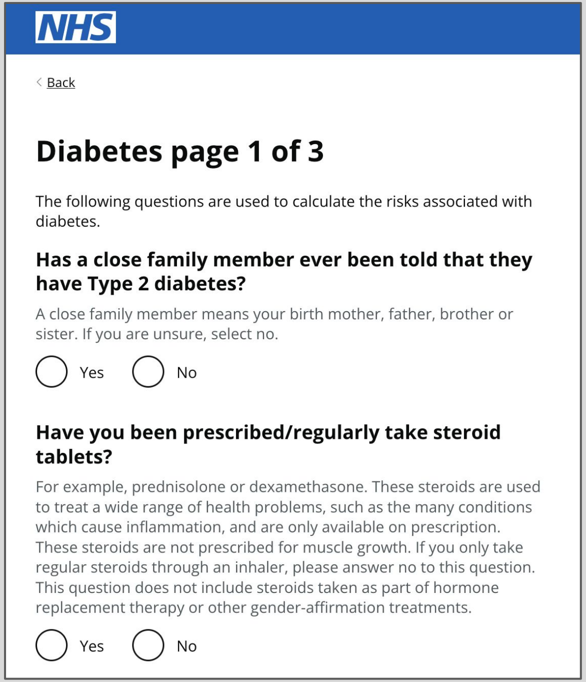
NHS

‹ Back

Diabetes page 1 of 3

The following questions are used to calculate the risks associated with diabetes.

Has a close family member ever been told that they have Type 2 diabetes?

A close family member means your birth mother, father, brother or sister. If you are unsure, select no.

○ Yes ○ No

Have you been prescribed/regularly take steroid tablets?

For example, prednisolone or dexamethasone. These steroids are used to treat a wide range of health problems, such as the many conditions which cause inflammation, and are only available on prescription. These steroids are not prescribed for muscle growth. If you only take regular steroids through an inhaler, please answer no to this question. This question does not include steroids taken as part of hormone replacement therapy or other gender-affirmation treatments.

○ Yes ○ No

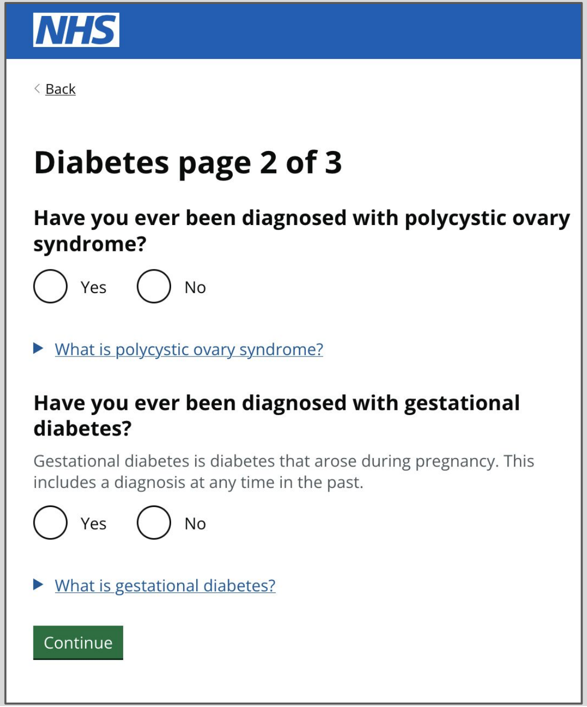
NHS

‹ Back

Diabetes page 2 of 3

Have you ever been diagnosed with polycystic ovary syndrome?

○ Yes ○ No

▶ What is polycystic ovary syndrome?

Have you ever been diagnosed with gestational diabetes?

Gestational diabetes is diabetes that arose during pregnancy. This includes a diagnosis at any time in the past.

○ Yes ○ No

▶ What is gestational diabetes?

Continue

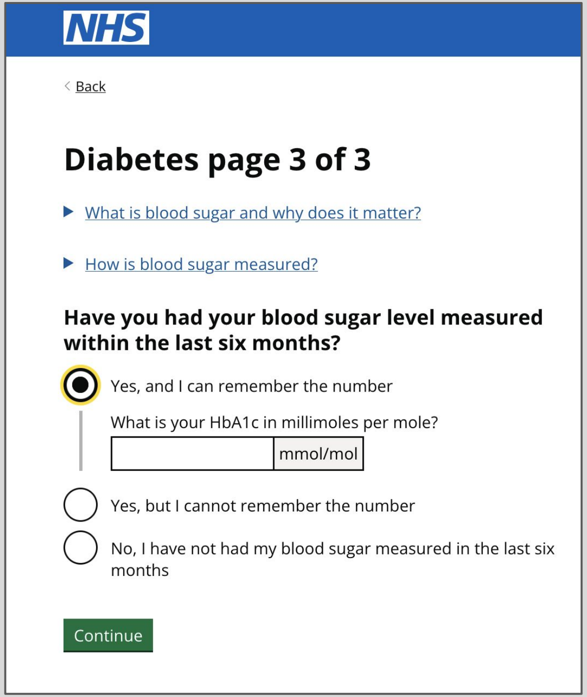
NHS

‹ Back

Diabetes page 3 of 3

▶ What is blood sugar and why does it matter?

▶ How is blood sugar measured?

Have you had your blood sugar level measured within the last six months?

◉ Yes, and I can remember the number

What is your HbA1c in millimoles per mole?

[ ] mmol/mol

○ Yes, but I cannot remember the number

○ No, I have not had my blood sugar measured in the last six months

Continue

Questions: diabetes risk factors, blood sugar

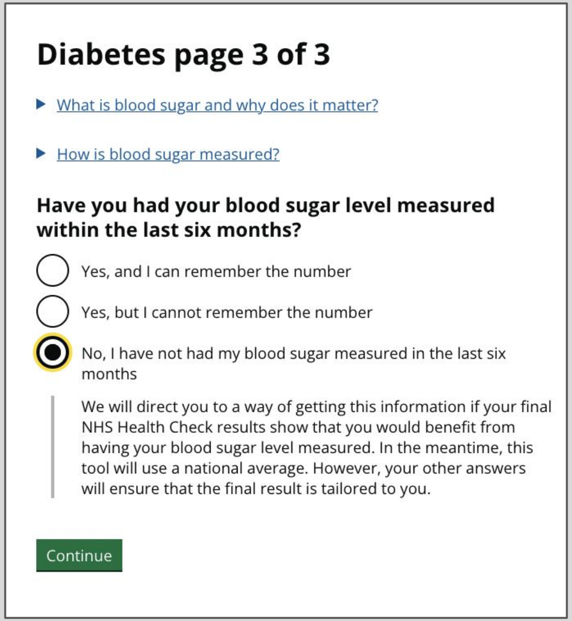

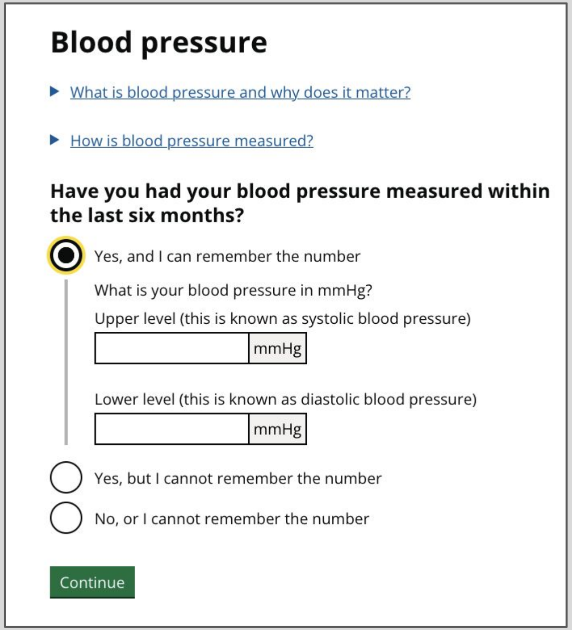

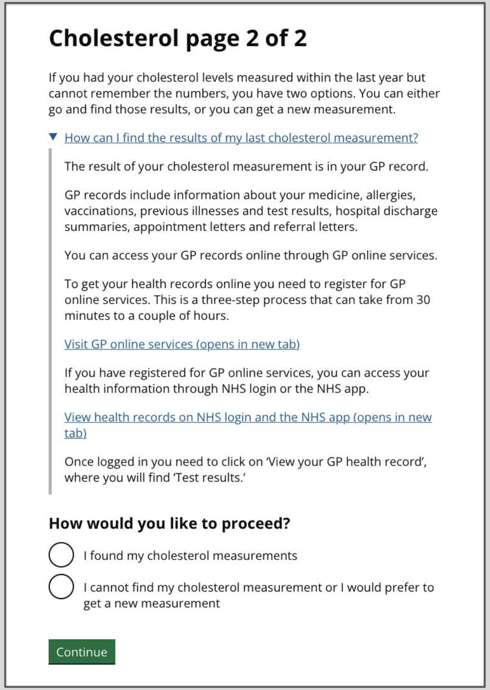

Biometrics options

For each biometric, there are different paths. If the service user knows their measurements, they can input them directly. If the service user has not had measurements recently, they will be offered ways to get them. If the service user has had their measurements taken recently but cannot remember or find them, they are offered ways to find them.

Risk factors page 1 of 3

The following factors influence your risk of heart and circulatory disease. If you don’t know, select no.

Has a close family member ever been told that they have angina or had a heart attack under the age of 60?

A close family member includes a birth mother, father, sister, or brother.

○ Yes ○ No

▶ What is angina?

Have you ever been diagnosed with chronic kidney disease?

Chronic kidney disease includes stage 3, 4, or 5 kidney disease.

○ Yes ○ No

▶ What is chronic kidney disease?

Have you ever been diagnosed with atrial fibrillation?

○ Yes ○ No

Risk factors page 2 of 3

Are you on blood pressure treatment?

○ Yes ○ No

Have you ever been diagnosed with migraine?

○ Yes ○ No

▶ What is migraine?

▶ What is the difference between a headache and migraine?

Have you been diagnosed with rheumatoid arthritis?

○ Yes ○ No

▶ What is rheumatoid arthritis and what is the difference between rheumatoid arthritis and other types of arthritis?

Risk factors page 3 of 3

The following questions are optional but will make the results more accurate for you.

Have you been diagnosed with systemic lupus erythematosus (SLE)?

○ Yes

○ No

or

○ Prefer not to say

▶ What is systemic lupus erythematosus?

Have you been diagnosed with a severe mental illness?

This includes schizophrenia, bipolar disorder and moderate/severe depression.

○ Yes

○ No

or

○ Prefer not to say

Questions: CVD risk factors

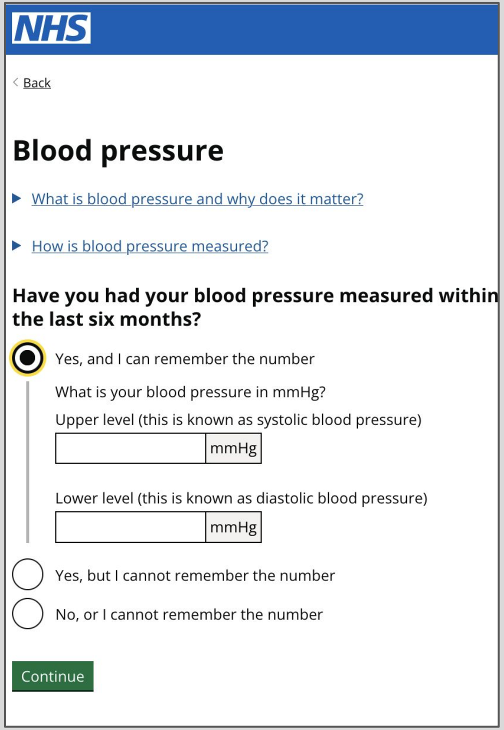

Biometrics: blood pressure

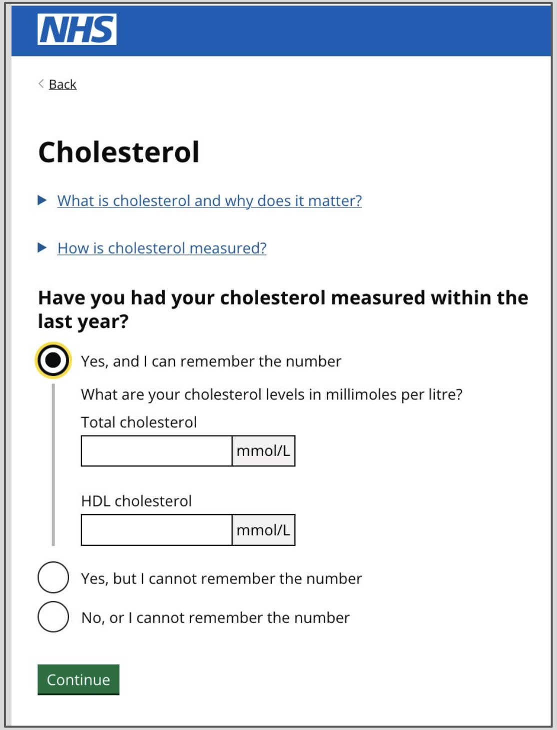

Biometrics: cholesterol

Mental wellbeing

You will now be asked questions that relate to your mental wellbeing. Your mental wellbeing influences your ability to choose, start and maintain a healthy lifestyle, and in so doing your risk of developing heart and circulatory disease, and Type 2 diabetes.

Looking after your mental wellbeing is important, not only when you are feeling low, anxious or stressed, but is something that plays a part in your everyday life.

Your responses to the questions below will be used to determine whether you might benefit from support for your mental wellbeing. If you select ‘Improve my mental wellbeing’ as one of your health priorities, you will be provided with web links to helpful mental wellbeing resources and services that are available in Southwark.

The next questions are to explore how you are feeling recently. It is understandable that you might experience anxiety or worry sometimes, depending on the situation. We are interested in if these feelings are serious enough to have a marked impact on your daily life.

If you do not want to use this digital NHS Health Check service to address your mental wellbeing, feel free to click the “Skip” button at the bottom of this page.

▸ If you do not want to use this digital NHS Health Check service to address your mental wellbeing, please read the information below.

During the past month, have you been bothered by feeling down, depressed or hopeless?

○ Yes ○ No

During the past month, have you often been bothered by having little interest or pleasure in doing things?

○ Yes ○ No

Over the last two weeks, how often have you been bothered by the following problems?

Feeling nervous, anxious or on edge

○ Not at all
○ Several days
○ More than half the days
○ Nearly every day

Not being able to stop or control worrying

○ Not at all
○ Several days
○ More than half the days
○ Nearly every day

Are you currently under the care of a psychologist or doctor for mental health problems?

○ Yes ○ No

Questions: mental wellbeing

Your communication preferences

Your results

As this is a medical assessment, your results will automatically be shared with your GP clinic. If your results show that you have a high risk of developing either Type 2 diabetes or heart and circulatory disease, please contact your GP clinic for a non-urgent discussion of your results. If your situation requires urgent action, the service will use the words 'urgent or urgently'.

You can also receive the results of your assessment via email. This will include a link that will allow you to add in new blood pressure, cholesterol or blood sugar results and get an updated assessment.

Follow-ups and notifications

The second part of this NHS Health Check service also offers follow-up advice and support to help you make steps to improve your health.

To receive the above mentioned results and follow-up support from this service, please provide your email below.

We may also use your email address and UK mobile telephone number to invite you to feedback your experience using the Digital NHS Health Check.

Your UK telephone number will be used if you later opt in to a telephone-based partner service. Your UK mobile phone number will be used if you later opt in for reminders.

How would you like to be contacted? (optional)

Select all options that are relevant to you.

- [ ] Email
- [ ] Phone
- [ ] Text message

Continue

Validation

This step allows us to share your results with your GP clinic.

What is your postcode?

Enter the postcode of the address where you live. This influences your risk score.

EC2Y 8DS

What is your first name?

This is used for personalisation.

Jean-Michel

What is your surname?

This is used for validation.

Basquiat

What is your date of birth?

This influences your risk score and is used for verification purposes. For example, 27 3 1982

Day 22 Month 12 Year 1960

Continue

Check your answers

Your results will be calculated on the next page based on the responses you have entered

You can go back and change any of your results by reviewing and amending the details below, but if you do not need to make any changes please scroll to the bottom of the page and submit your measurements.

Your answers

About you

Your personal details

**Your date of birth**	22/12/1960	Change
**Your postcode**	EC2Y8DS	Change
**Your height**	6 feet 0 inches	Change
**Your weight**	11 stone 10 pounds	Change
**Your sex assigned at birth**	Male	Change
**Your current gender identity**	Same as my sex assigned at birth	Change
**Your ethnicity**	Caribbean	Change

Contact preferences, validation, check answers

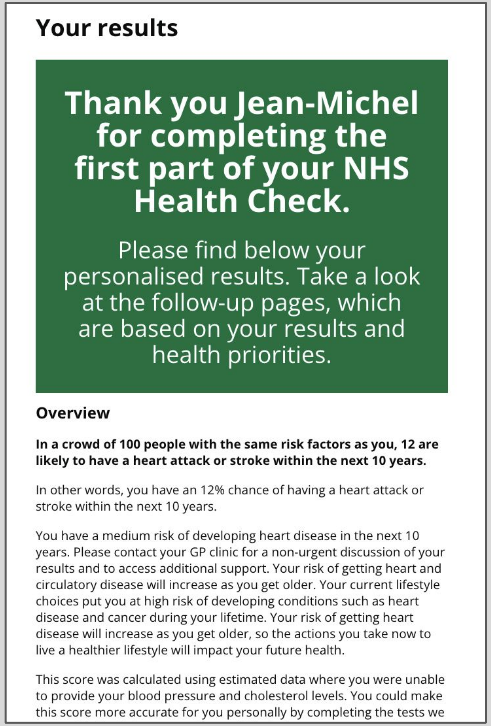
Your results

Thank you Jean-Michel for completing the first part of your NHS Health Check.

Please find below your personalised results. Take a look at the follow-up pages, which are based on your results and health priorities.

Overview

In a crowd of 100 people with the same risk factors as you, 12 are likely to have a heart attack or stroke within the next 10 years.

In other words, you have an 12% chance of having a heart attack or stroke within the next 10 years.

You have a medium risk of developing heart disease in the next 10 years. Please contact your GP clinic for a non-urgent discussion of your results and to access additional support. Your risk of getting heart and circulatory disease will increase as you get older. Your current lifestyle choices put you at high risk of developing conditions such as heart disease and cancer during your lifetime. Your risk of getting heart disease will increase as you get older, so the actions you take now to live a healthier lifestyle will impact your future health.

This score was calculated using estimated data where you were unable to provide your blood pressure and cholesterol levels. You could make this score more accurate for you personally by completing the tests we

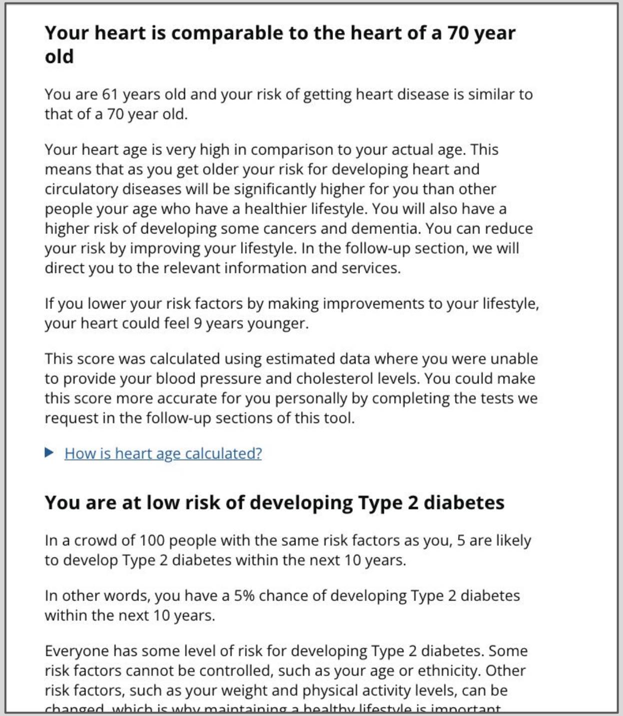
Your heart is comparable to the heart of a 70 year old

You are 61 years old and your risk of getting heart disease is similar to that of a 70 year old.

Your heart age is very high in comparison to your actual age. This means that as you get older your risk for developing heart and circulatory diseases will be significantly higher for you than other people your age who have a healthier lifestyle. You will also have a higher risk of developing some cancers and dementia. You can reduce your risk by improving your lifestyle. In the follow-up section, we will direct you to the relevant information and services.

If you lower your risk factors by making improvements to your lifestyle, your heart could feel 9 years younger.

This score was calculated using estimated data where you were unable to provide your blood pressure and cholesterol levels. You could make this score more accurate for you personally by completing the tests we request in the follow-up sections of this tool.

▸ How is heart age calculated?

You are at low risk of developing Type 2 diabetes

In a crowd of 100 people with the same risk factors as you, 5 are likely to develop Type 2 diabetes within the next 10 years.

In other words, you have a 5% chance of developing Type 2 diabetes within the next 10 years.

Everyone has some level of risk for developing Type 2 diabetes. Some risk factors cannot be controlled, such as your age or ethnicity. Other risk factors, such as your weight and physical activity levels, can be changed, which is why maintaining a healthy lifestyle is important

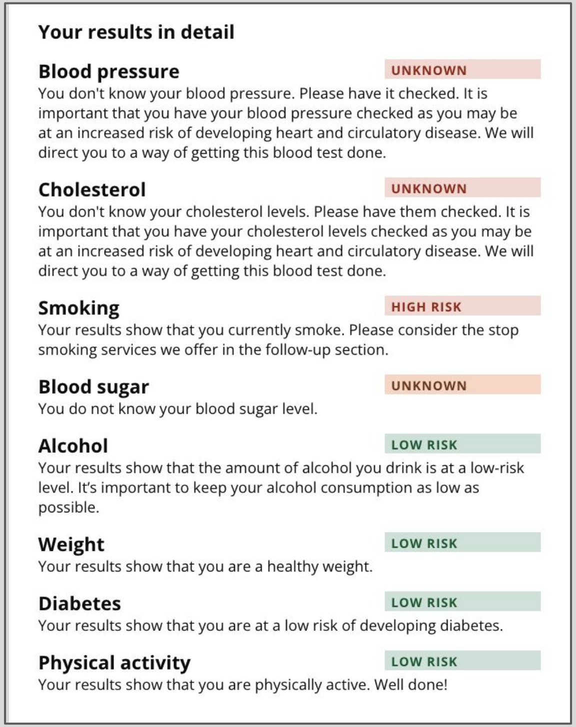
Your results in detail

Blood pressure UNKNOWN
You don't know your blood pressure. Please have it checked. It is important that you have your blood pressure checked as you may be at an increased risk of developing heart and circulatory disease. We will direct you to a way of getting this blood test done.

Cholesterol UNKNOWN
You don't know your cholesterol levels. Please have them checked. It is important that you have your cholesterol levels checked as you may be at an increased risk of developing heart and circulatory disease. We will direct you to a way of getting this blood test done.

Smoking HIGH RISK
Your results show that you currently smoke. Please consider the stop smoking services we offer in the follow-up section.

Blood sugar UNKNOWN
You do not know your blood sugar level.

Alcohol LOW RISK
Your results show that the amount of alcohol you drink is at a low-risk level. It's important to keep your alcohol consumption as low as possible.

Weight LOW RISK
Your results show that you are a healthy weight.

Diabetes LOW RISK
Your results show that you are at a low risk of developing diabetes.

Physical activity LOW RISK
Your results show that you are physically active. Well done!

Results

Each service user gets a personalised results page, including an overview, their QRISK explained in a few different ways, comparable heart age, Qdiabetes, mental health summary, and then results in detail to highlight the suggested actions.

GP Report Summary

The following is the existing Digital Health Check's GP report, as sent via email to GP practices on completion of a digital health check (with test data).

Dear {name of surgery, for example, Elm Lodge} GP Surgery,

Re: Digital NHS Health Check results

Your patient has completed their NHS Health Check, using Southwark's digital NHS Health Check tool. This email includes: your patient's CVD and Type 2 diabetes risk scores and heart age; recommendations for clinical follow-up and referral-on; and a full breakdown of their results.

We recommend uploading this data into your patient's record. To support this, relevant SNOMED codes are included. Southwark's digital NHS Health Check has been developed by Southwark Council's public health team with our IT partner QMS ["QMS website"], and designed in partnership with eligible residents and local clinicians. The tool uses licensed medical devices QRisk3 and QDiabetes to assess an individual's risk of developing cardiovascular disease, Type 2 diabetes and healthy heart age. This is the same method as a face-to-face assessment. If you have any questions about the digital NHS Health Check please contact Southwark Council's public health team on publichealth@southwark.gov.uk

Registered patient: {Patient12 Surname}
DOB: 01/04/1975 **Postcode:** BH12EF **NHS Number:** 6850120012

Risk score:
QRISK3 Cardiovascular disease 10-year risk of developing CVD: {number}, SNOMED code: {number}
Lifetime risk of developing CVD: {number}, SNOMED code: {number}
10-year risk of developing Type 2 diabetes: {number}, SNOMED code: {number}

Heart Age:
Healthy heart age is comparable to that of an individual aged: {number}, SNOMED code: {number}

Follow-up requirements:
{If follow-up required} Your patient has the following elevated risks requiring clinical follow-up or referral:

{Include if Amber or Red}10-year risk of developing CVD: {number},
{Include if Amber or Red} Lifetime risk of developing CVD: {number},

Full breakdown of results are below

Situation: 840391000000101 | National Health Service Health Check completed by third party

Age: {value}

Sex assigned at birth: {value}

Gender identity: {value}

Sex used to calculate risk scores: {value}

Smoking Status: {value}, 160618006 | Current non-smoker (finding)

Smoking Status: {value}, 8517006 | Ex-smoker (finding)

Smoking Status: {value}, 160603005 | Light cigarette smoker (1-9 cigs/day) (finding)

Smoking Status: {value}, 160604004 | Moderate cigarette smoker (10-19 cigs/day) (finding)

Smoking Status: {value}, 160605003 | Heavy cigarette smoker (20-39 cigs/day) (finding)

Family History of CVD: {value}, 266895004 | Family history: Ischemic heart disease at less than 60 years (situation)

Family History of CVD: {value}, 266882009 | No family history: Ischemic heart disease (situation)

Ethnicity: {value}, 976631000000101 | White: English, Welsh, Scottish, Northern Irish or British – England and Wales ethnic category 2011 census (finding)

Ethnicity: {value}, 976651000000108 | White: Irish – England and Wales ethnic category 2011 census (finding)

Ethnicity: {value}, 976671000000104 | White: Gypsy or Irish Traveller – England and Wales ethnic category 2011 census (finding)

Ethnicity: {value}, 976691000000100 | White: any other White background – England and Wales ethnic category 2011 census (finding)

Ethnicity: {value}, 976711000000103 | Mixed multiple ethnic groups: White and Black Caribbean – England and Wales ethnic category 2011 census (finding)

Ethnicity: {value}, 976731000000106 | Mixed multiple ethnic groups: White and Black African – England and Wales ethnic category 2011 census (finding)

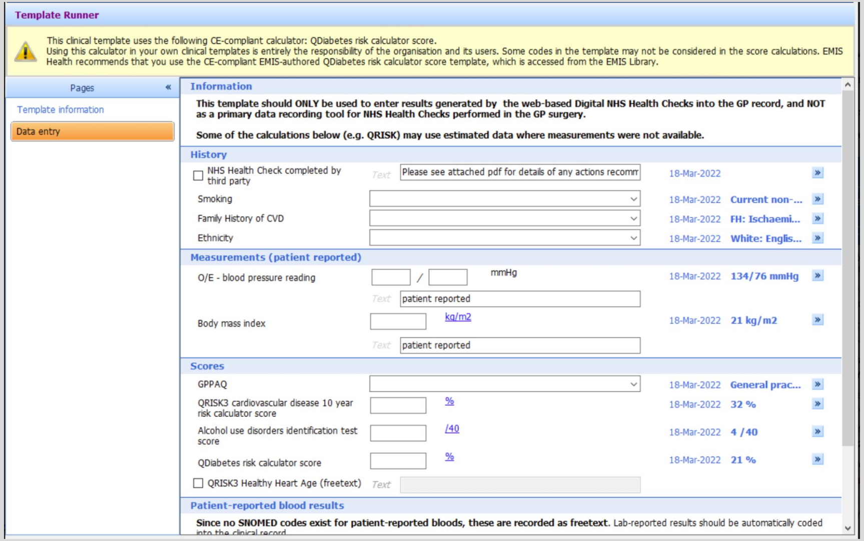

GP report summary via email, EMIS template

This GP report is automatically sent to the GP surgery once the citizen calculates their results. We also created an EMIS template for the DHC, which can be uploaded to each practice's system to expedite the upload of information, rather than having to code each value individually.

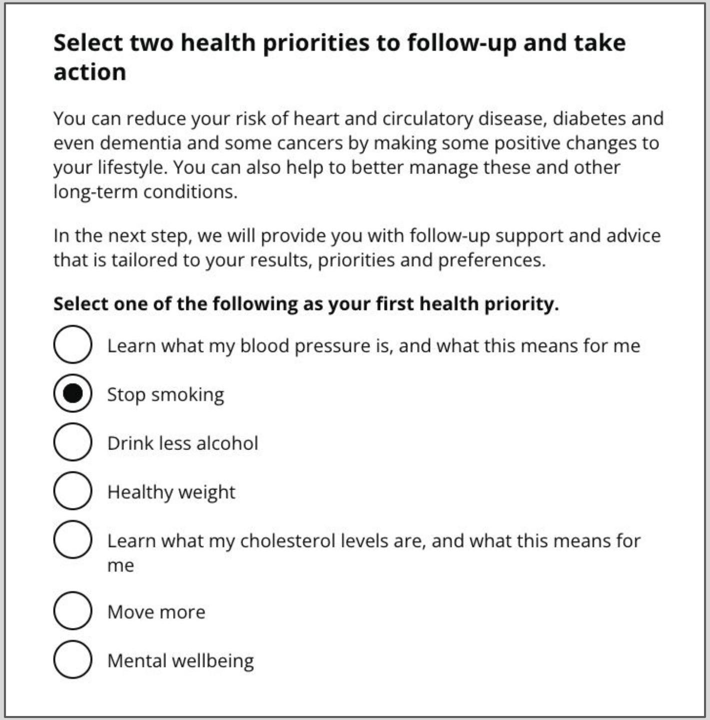

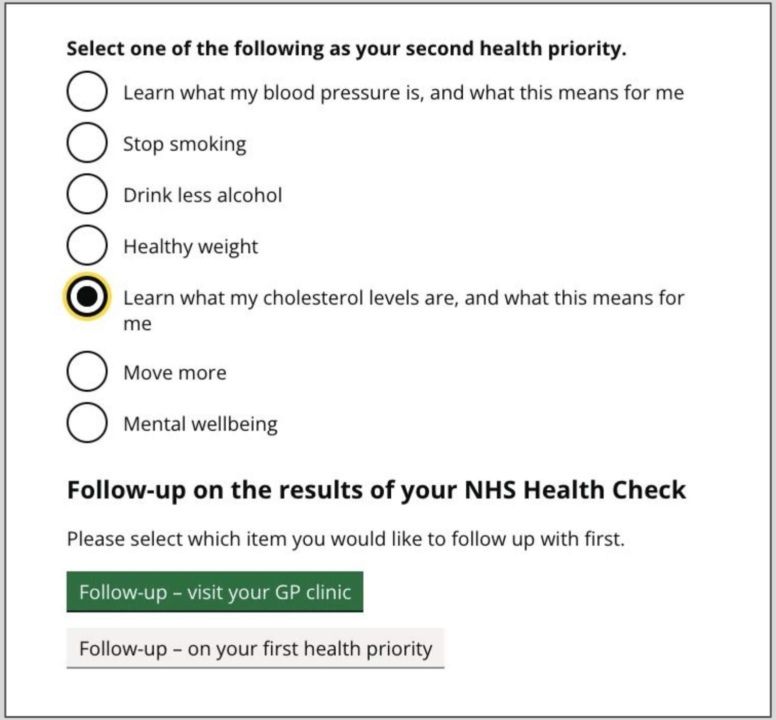

Health priorities

At the bottom of the results page, after the explanations of their risk, service users are recommended to select two priorities of actions they would like to take. If they are above a given, risk threshold, they are shown the option to follow up with their GP. In any case, they can then follow up on their health priorities.

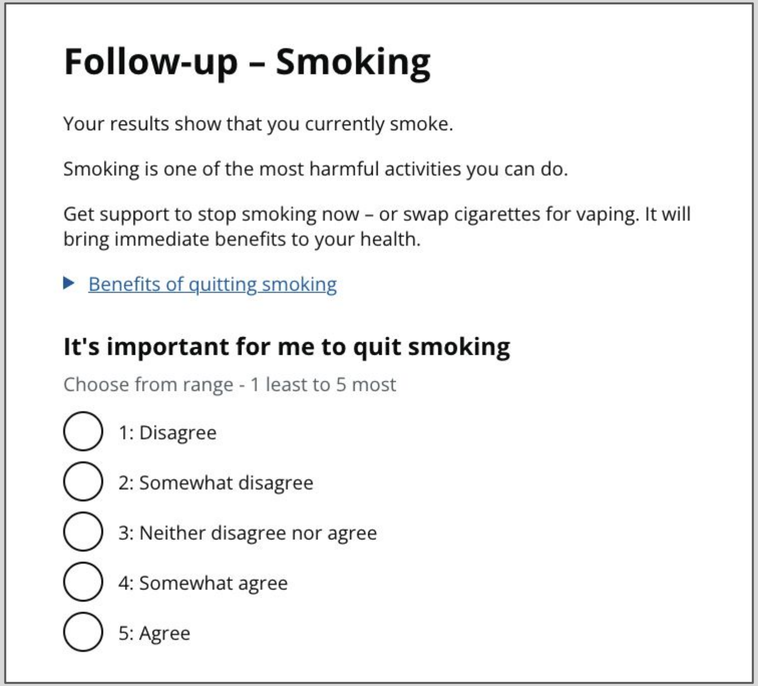
Follow-up – Smoking

Your results show that you currently smoke.

Smoking is one of the most harmful activities you can do.

Get support to stop smoking now – or swap cigarettes for vaping. It will bring immediate benefits to your health.

▶ Benefits of quitting smoking

It's important for me to quit smoking

Choose from range - 1 least to 5 most

- ◯ 1: Disagree
- ◯ 2: Somewhat disagree
- ◯ 3: Neither disagree nor agree
- ◯ 4: Somewhat agree
- ◯ 5: Agree

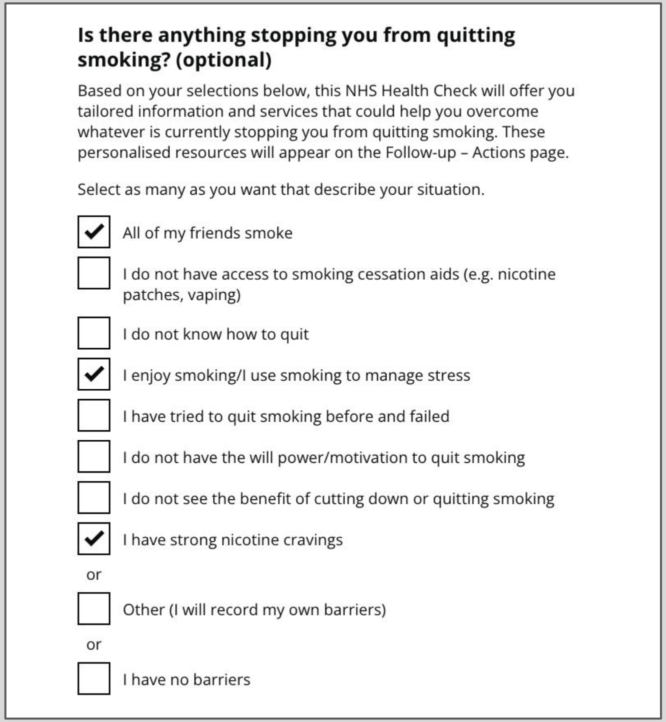
Is there anything stopping you from quitting smoking? (optional)

Based on your selections below, this NHS Health Check will offer you tailored information and services that could help you overcome whatever is currently stopping you from quitting smoking. These personalised resources will appear on the Follow-up – Actions page.

Select as many as you want that describe your situation.

- [x] All of my friends smoke
- [ ] I do not have access to smoking cessation aids (e.g. nicotine patches, vaping)
- [ ] I do not know how to quit
- [x] I enjoy smoking/I use smoking to manage stress
- [ ] I have tried to quit smoking before and failed
- [ ] I do not have the will power/motivation to quit smoking
- [ ] I do not see the benefit of cutting down or quitting smoking
- [x] I have strong nicotine cravings

or

- [ ] Other (I will record my own barriers)

or

- [ ] I have no barriers

Follow up: lifestyle behaviour change, barriers

Each “Follow up” page follows the same format, checking how important that change is for the person and helping them to consider their personal barriers to making that change, so that they can be matched with an intervention that corresponds to their individual needs.

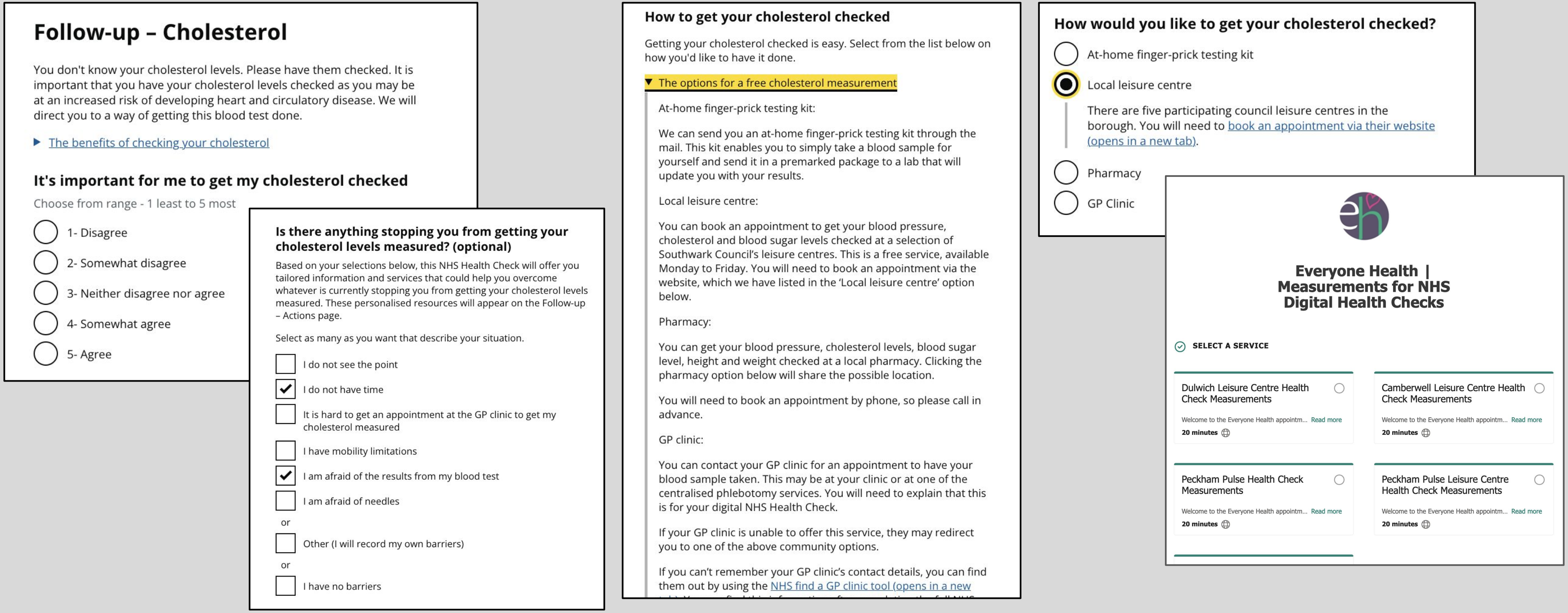

Follow-up: biometrics options and barriers

In addition to the basic questions about what barriers are stopping the individual from completing the biometric measurement, they are offered options to get the measurement done in the community: at home, leisure centre, pharmacy, kiosk or GP clinic. They can then select which option works best for them and make the corresponding arrangements.

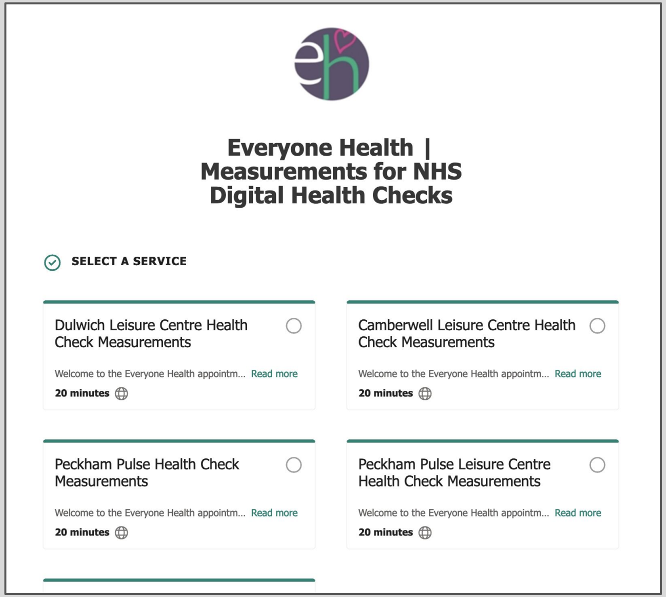

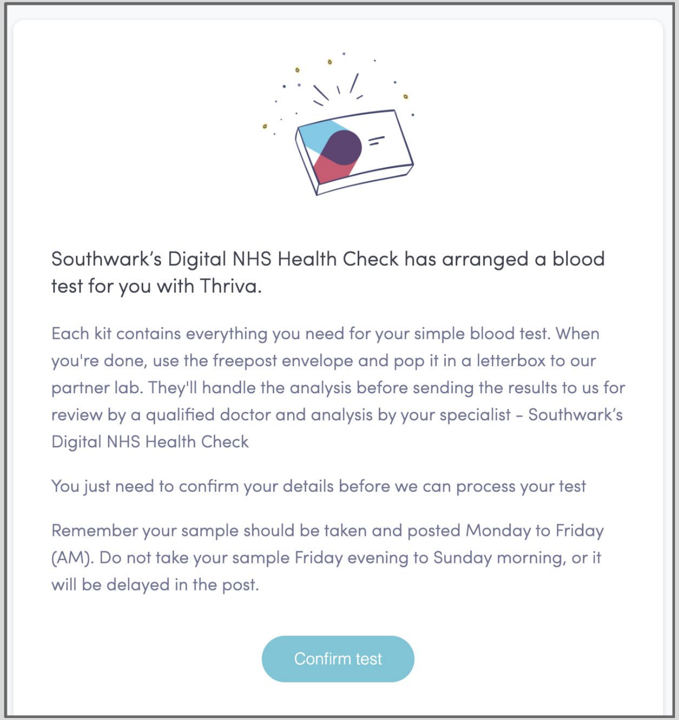

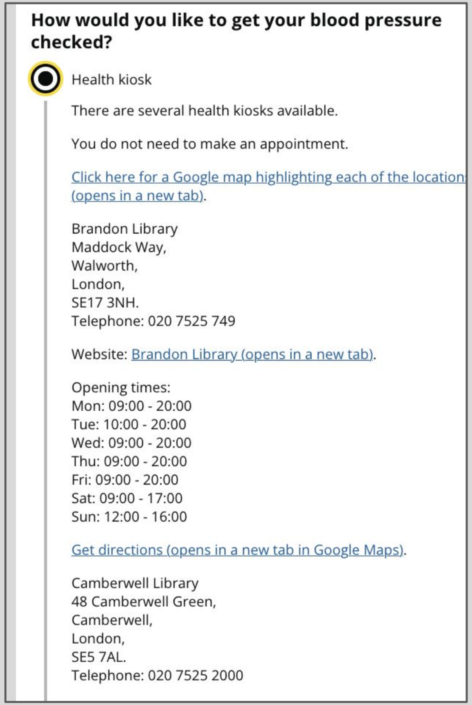

Biometrics example: link to scheduling, sign-up email for blood kit, addresses for kiosk

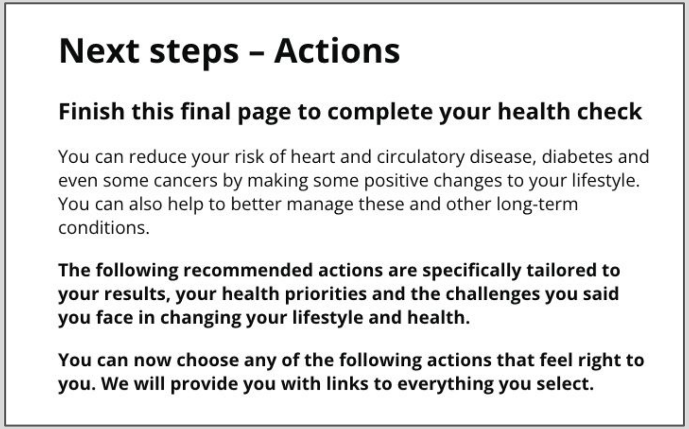

Next steps – Actions

Finish this final page to complete your health check

You can reduce your risk of heart and circulatory disease, diabetes and even some cancers by making some positive changes to your lifestyle. You can also help to better manage these and other long-term conditions.

The following recommended actions are specifically tailored to your results, your health priorities and the challenges you said you face in changing your lifestyle and health.

You can now choose any of the following actions that feel right to you. We will provide you with links to everything you select.

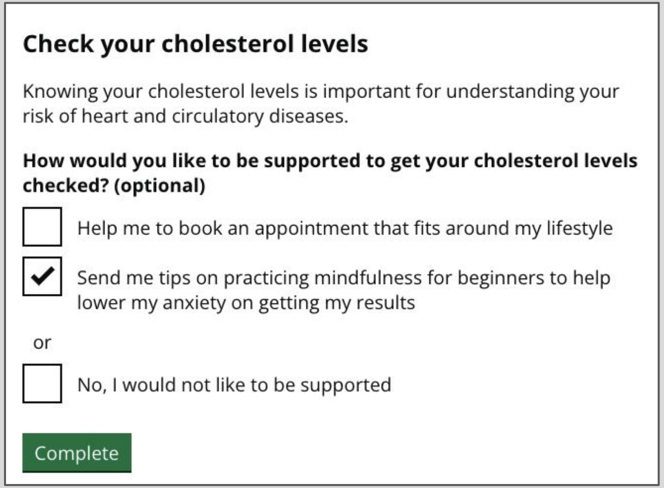

Check your cholesterol levels

Knowing your cholesterol levels is important for understanding your risk of heart and circulatory diseases.

How would you like to be supported to get your cholesterol levels checked? (optional)

- [ ] Help me to book an appointment that fits around my lifestyle
- [x] Send me tips on practicing mindfulness for beginners to help lower my anxiety on getting my results

or

- [ ] No, I would not like to be supported

Complete

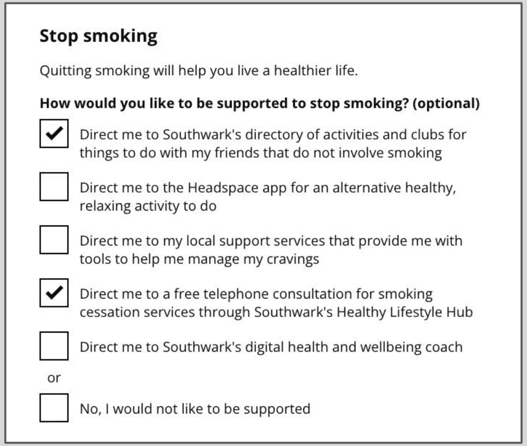

Stop smoking

Quitting smoking will help you live a healthier life.

How would you like to be supported to stop smoking? (optional)

- [x] Direct me to Southwark's directory of activities and clubs for things to do with my friends that do not involve smoking
- [ ] Direct me to the Headspace app for an alternative healthy, relaxing activity to do
- [ ] Direct me to my local support services that provide me with tools to help me manage my cravings
- [x] Direct me to a free telephone consultation for smoking cessation services through Southwark's Healthy Lifestyle Hub
- [ ] Direct me to Southwark's digital health and wellbeing coach

or

- [ ] No, I would not like to be supported

Follow up: Actions and interventions

Based on their individual health needs and the barriers selected by the service user in the “Follow up” pages, they are offered personalised suggested actions and interventions that correspond directly to these barriers.

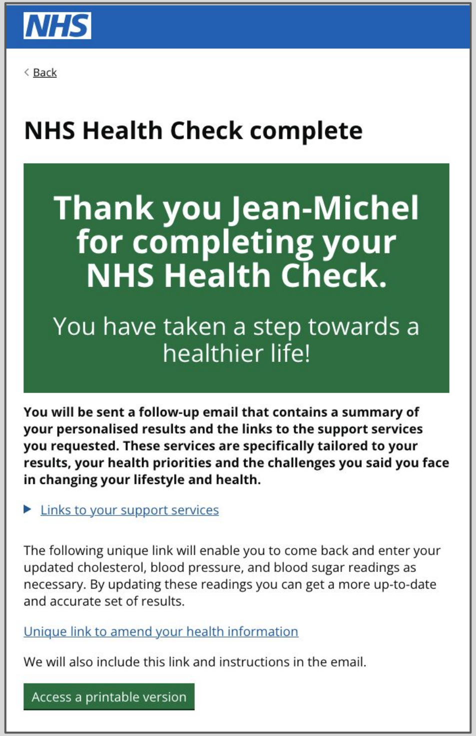

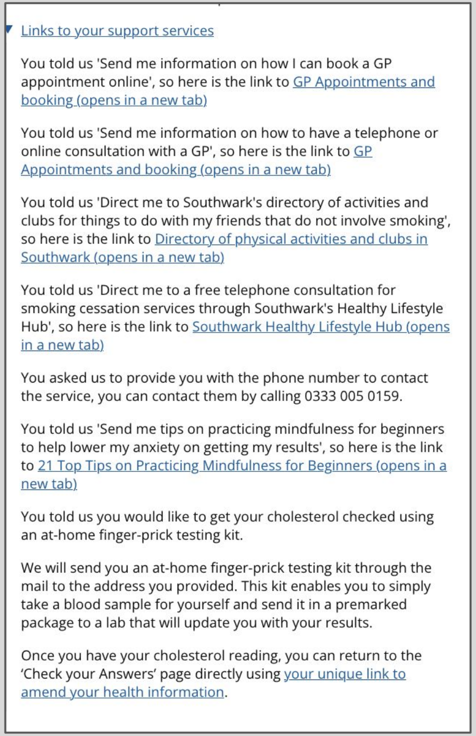

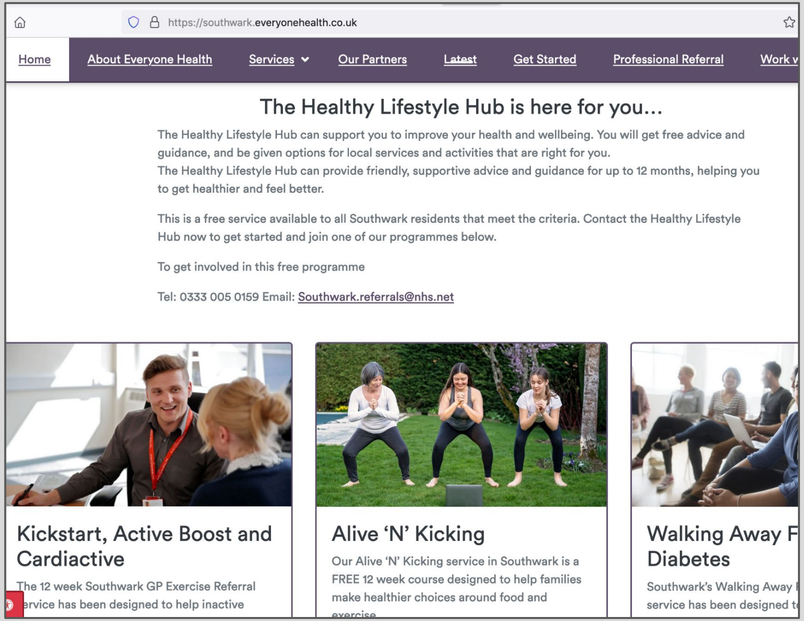

Complete page, support services

The "Complete" page offers direct links to the Support services/interventions the service user requested and a printable version of their results. It also offers a unique link to amend their information if for example they get new biometrics measurements. If they do input new measurements, they can get updated results, recommendations and interventions.

2 of 9,

Digital NHS Health Check Results - PATIENT SUMMARY Inbox ×

NHS Digital Health Check noreply.quicksilva@nhs.uk via nhs.uk 10:58 PM (3 minutes ago)
to me

This service is currently in development and testing. Please share any feedback in the survey.

Your Digital NHS Health Check: Results 29/05/2022

Hello

Thank you for completing your NHS Health Check, the medical service that assesses your risk of developing heart and circulatory diseases as well as Type 2 diabetes. This service helps you to take control of your health and wellbeing.

Your feedback is important to us. It can help to shape our new Digital NHS Health Check service and ultimately help improve the health and wellbeing of others like yourself.

There will be three short surveys across three months – starting with the one below. Complete all three and be entered into a prize draw to win Decathlon gift vouchers!

Your support services:

These services are specifically tailored to your results, your health priorities and the challenges you said you face in changing your lifestyle and health.

You told us 'Send me information on how I can book a GP appointment online', so here is the link to GP Appointments and booking

You told us 'Send me information on how to have a telephone or online consultation with a GP', so here is the link to GP Appointments and booking

You told us 'Direct me to Southwark's directory of activities and clubs for things to do with my friends that do not involve smoking', so here is the link to Directory of physical activities and clubs in Southwark

You told us 'Direct me to a free telephone consultation for smoking cessation services through Southwark's Healthy Lifestyle Hub', so here is the link to Southwark Healthy Lifestyle Hub

You asked us to provide you with the phone number to contact the service, you can contact them by calling 0333 005 0159.

You told us 'Send me tips on practicing mindfulness for beginners to help lower my anxiety on getting my results', so here is the link to 21 Top Tips on Practicing Mindfulness for Beginners

You told us you would like to get your cholesterol checked using an at-home finger-prick testing kit.

Your results:

In a crowd of 100 people with the same risk factors as you, 12 are likely to have a heart attack or stroke within the next 10 years.

In other words, you have a 12% chance of having a heart attack or stroke within the next 10 years.

You have a medium risk of developing heart disease in the next 10 years. Please contact your GP clinic for a non-urgent discussion of your results and to access additional support. Your risk of getting heart and circulatory disease will increase as you get older. Your current lifestyle choices put you at high risk of developing conditions such as heart disease and cancer during your lifetime. Your risk of getting heart disease will increase as you get older, so the actions you take now to live a healthier lifestyle will impact your future health.

This score was calculated using estimated data where you were unable to provide your blood pressure and cholesterol levels. You could make this score more accurate for you personally by completing the tests we request in the follow-up sections of this tool.

Results email

The service user also receives an email with a copy of their results, links to their requested support services/interventions, and the unique link to their questionnaire so they can update it with new biometrics and receive new results, recommendations, and interventions.
